# Unbinding Kinetics of Muscarinic M3 Receptor Antagonists Explained by Metadynamics Simulations

**DOI:** 10.1021/acs.jcim.3c00042

**Published:** 2023-04-13

**Authors:** Francesca Galvani, Daniele Pala, Alberto Cuzzolin, Laura Scalvini, Alessio Lodola, Marco Mor, Andrea Rizzi

**Affiliations:** † Dipartimento di Scienze degli Alimenti e del Farmaco, Università degli Studi di Parma, Parco Area delle Scienze 27/A, I-43124 Parma, Italy; ‡ Chemistry Research and Drug Design Department, Chiesi Farmaceutici S.p.A., Largo F. Belloli 11/A, 43122 Parma, Italy; § Microbiome Research Hub, University of Parma, Parco Area delle Scienze 11/A, I-43124 Parma, Italy

## Abstract

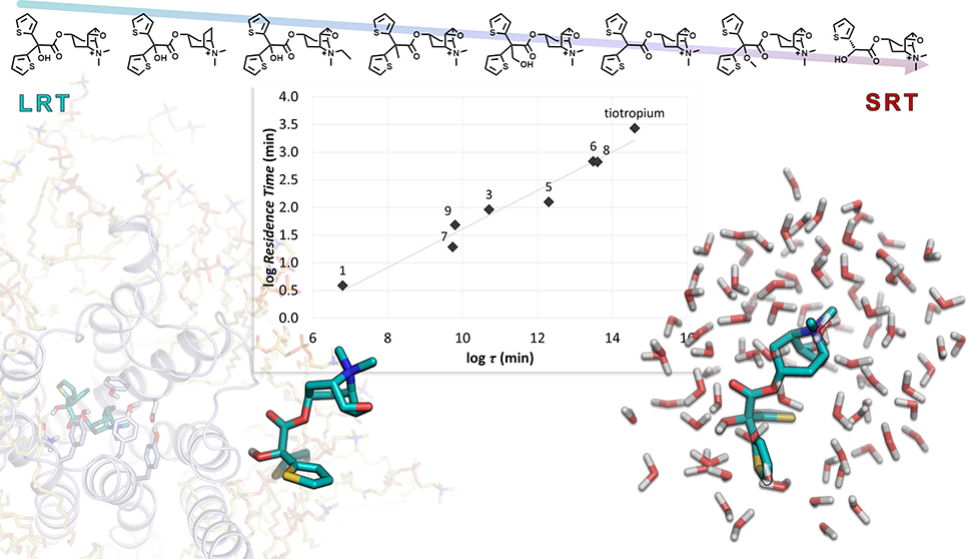

The residence time (RT), the time for which a drug remains bound to its biological target, is a critical parameter for drug design. The prediction of this key kinetic property has been proven to be challenging and computationally demanding in the framework of atomistic simulations. In the present work, we setup and applied two distinct metadynamics protocols to estimate the RTs of muscarinic M3 receptor antagonists. In the first method, derived from the conformational flooding approach, the kinetics of unbinding is retrieved from a physics-based parameter known as the acceleration factor α (i.e., the running average over time of the potential deposited in the bound state). Such an approach is expected to recover the absolute RT value for a compound of interest. In the second method, known as the *t*_META-D_ approach, a qualitative estimation of the RT is given by the time of simulation required to drive the ligand from the binding site to the solvent bulk. This approach has been developed to reproduce the change of experimental RTs for compounds targeting the same target. Our analysis shows that both computational protocols are able to rank compounds in agreement with their experimental RTs. Quantitative structure–kinetics relationship (SKR) models can be identified and employed to predict the impact of a chemical modification on the experimental RT once a calibration study has been performed.

## Introduction

**Figure 1:**
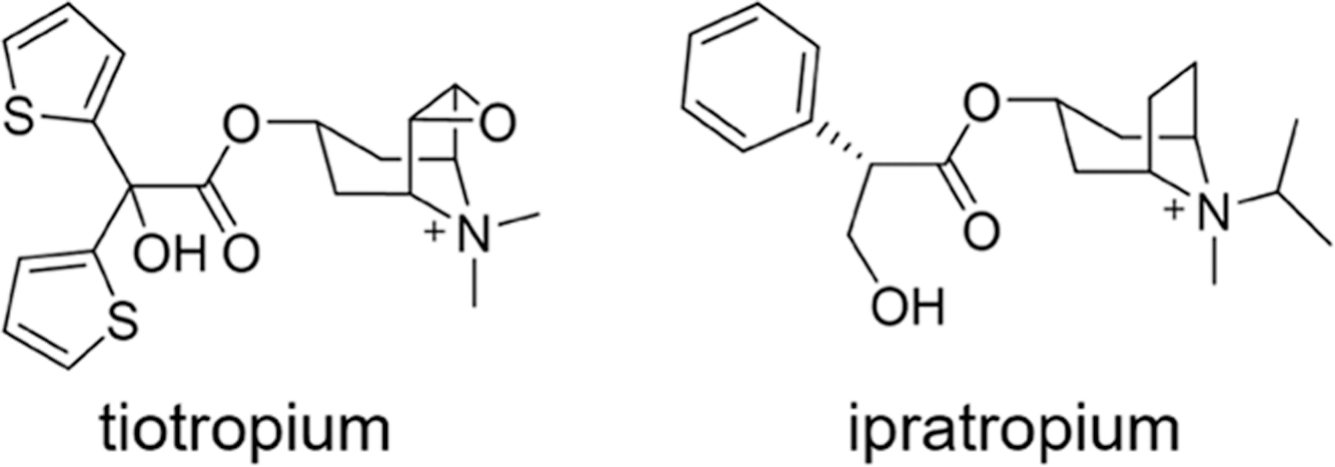
Chemical structures of the clinically approved M3 antagonists tiotropium and ipratropium.

Among the different parameters dictating the pharmacological effect of a small-molecule drug agent, the residence time (RT) at a protein target has recently gained attention as an important factor for predicting the duration of action and efficacy *in vivo*.^1^ The concentration of a drug fluctuates over time by virtue of a dynamic flow *in vivo* due to the well-known phases of pharmacokinetics, i.e., absorption, distribution, metabolism, and excretion. In this context, equilibrium conditions are far from being satisfied,^2,3^ with kinetic properties such as RT or its reciprocal, the dissociation rate constant (*k*_off_), being equally informative in terms of biological activity of a drug as thermodynamic properties, like the binding affinity (*K*_D_) or the inhibitory (*k*_*i*_) constants. Optimization of the RT parameter has emerged as an effective strategy to control key drug properties, such as duration of action,^4^ selectivity,^5^ and, most importantly, safety.^6−8^ The RT parameter has been actively used in discovery campaigns to guide the optimization of small-molecule compounds.^9^ One of the most representative programs based on the RT concept is the one aimed at the identification of long-acting muscarinic receptor antagonists, as in the case of the approved drug tiotropium (Figure 1).^10^ Thanks to a RT higher than 24 h, this compound can be dosed less frequently than the closely related antagonist ipratropium (Figure 1), which is instead featured by a RT of nearly 0.5 h.^11,12^

Although characterized by a similar equilibrium affinity for both muscarinic subtype receptors M2 and M3, tiotropium dissociates from M3 at a significantly slower rate than from M2, displaying kinetic selectivity *in vivo*.^11,12^ This and other similar scenarios have prompted the development of computational methods specifically focused on the prediction of RT,^13^ in some cases with the muscarinic receptors as reference systems.^14−17^

Computational approaches designed to describe ligand unbinding and build structure–kinetic relationships (SKRs) appear highly valuable in the context of lead-optimization campaigns, particularly if the experimental determination of RT is limited in its throughput. In principle, molecular dynamics (MD) is an ideal tool to estimate drug RT in light of its ability to give physical insights into protein–ligand interactions at an atomistic level.^8^ However, the experimental RT of several marketed drugs ranges from minutes to hours, and this time scale is far beyond the current possibilities of plain MD simulations.^18,19^ This limitation has pushed the development of MD-based approaches to accelerate the exploration of high-energy regions of the conformational landscape of protein–ligand complexes, allowing us to simulate the full unbinding in a reasonable computational time.^20^ Enhanced sampling methods have been largely used to rationalize SKRs of biologically active compounds,^21^ including steered molecular dynamics (SMD),^22^ adiabatic-bias MD (ABMD),^23^ smoothed-potential MD,^24^ weighted ensemble MD,^25^ supervised MD (SuMD),^26^ random-accelerated MD (RAMD),^27^ and metadynamics (META-D) simulations.^28^ In this context, we sought to setup and apply computational protocols able to describe ligand unbinding and to identify a meaningful energy-based descriptor that could rank compounds in agreement with their experimental RTs.

**Figure 2:**
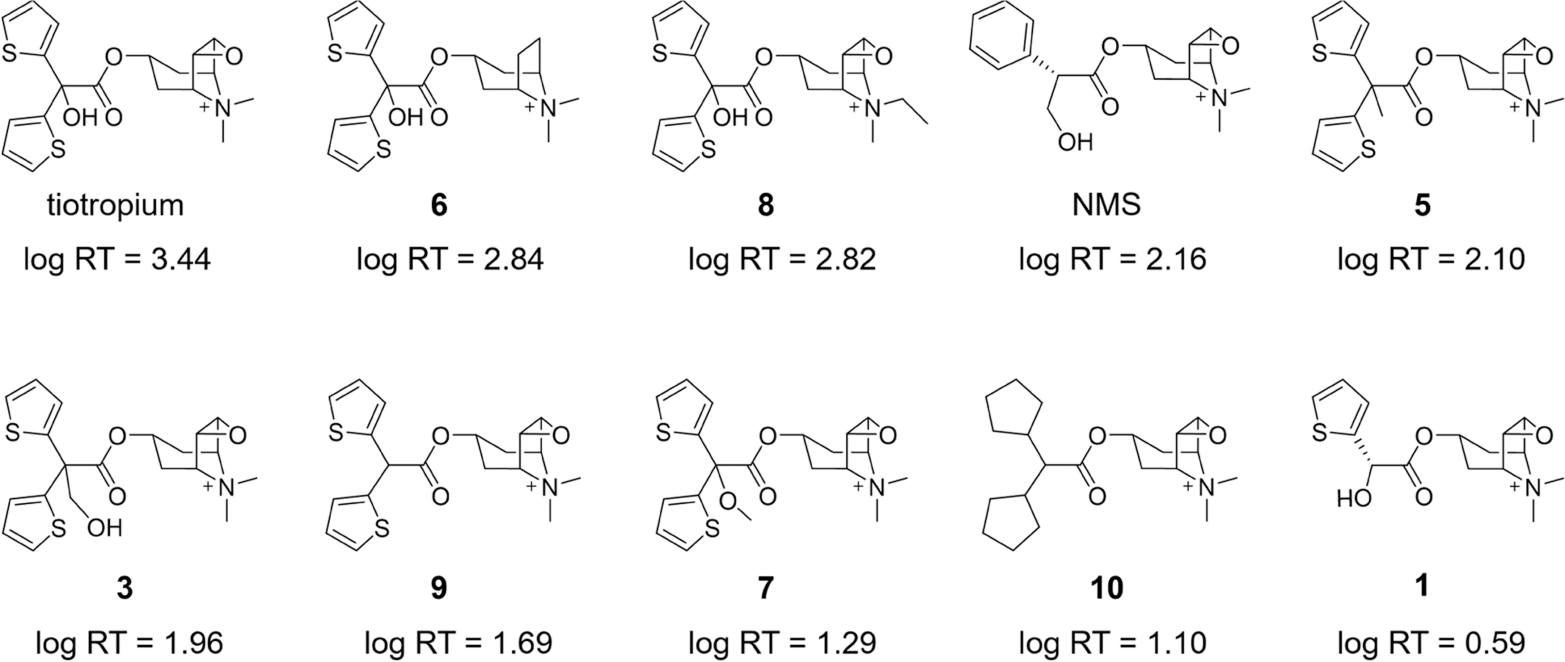
Chemical structures and experimental RT values (expressed in minutes, log unit) of the Tautermann data set of M3 antagonists^34^ ranked according to the experimental RT.

Considering our interest in SKRs on GPCR ligands,^29^ and, in particular, in M3 antagonists as single^30,31^ or dual targeting agents,^32,33^ we selected the M3 receptor as a reference system, focusing our attention on a set of tiotropium analogues for which experimental RTs have been recently reported by Tautermann and colleagues (Figure 2),^34^ and X-ray structures are currently available. Moderate modifications in the structure of tiotropium, including the removal of thiophene (**1**) or of an α-hydroxyl substituent (**9**), lead to a significant reduction in the experimental RT (over different orders of magnitude), suggesting that this set of compounds is suitable for the setup and test of computational protocols for SKR rationalization.

We started our investigation by applying a variant of the conformational flooding algorithm^35,36^ to describe the dissociation of tiotropium and related compounds from the M3 receptor. Under the assumption that the unbinding process can be simplified to a two-state model and no biasing potential is deposited in the transition state area separating bound and unbound states, this approach should allow us to retrieve the RT value of a ligand (RT_calcd_) as the product between the acceleration factor α^37^ and the transition time recorded in a well-tempered metadynamics (wt-META-D) simulation.^38^ As the unbinding process obeys the law of rare events,^39^ RT_calcd_ values were collected over a set of several wt-META-D simulations distributed according to a Poisson curve, with the characteristic parameter τ of this distribution representing the absolute RT of the compound of interest.^40^ After performing an optimization study aimed at the identification of suitable collective variables (CVs) for ligand unbinding, the computational parameter τ was retrieved for all of the compounds of the Tautermann data set, allowing an evaluation of the performance of the approach (in terms of accuracy and precision) and the search for a correlative SKR model between experimental and computed RT values.

We continued our work by testing the empirical *t*_META-D_ method that, differently from the conformational flooding algorithm, has been developed to reproduce variation of experimental RTs in response to chemical modifications for a set of compounds binding the same target.^41^ The *t*_META-D_ approach consists of performing META-D simulations of a protein–ligand system of interest depositing Gaussians of constant height (i.e., without wt rescaling) on CVs able to drive the unbinding process.^41^ A qualitative estimation of the RT is thus simply given by the time of a META-D simulation required to drive the ligand from the binding site to the solvent bulk. In our case, considering the uncertainty affecting this parameter, the *t*_META-D_ value was obtained by averaging the time of simulation of unbinding over several independent META-D simulations. Finally, averaged *t*_META-D_ parameters were calculated for all of the compounds of the M3 data set, and a correlative model was built and compared to that obtained with the conformational flooding algorithm.

Anticipating our results, we show here the ability of metadynamics simulations to provide computed parameters suitable for the identification of quantitative SKR equations able to provide a fair estimation of experimental RTs. Once a calibration study has been properly performed, SKR models of this kind could be applied to predict the impact of a chemical modification on the experimental RT in the context of ligand optimization, being thus useful for drug design.

## Results

### Molecular Models of Human M3 (*h*M3) in a Complex with Tiotropium-Derived Antagonists

**Figure 3:**
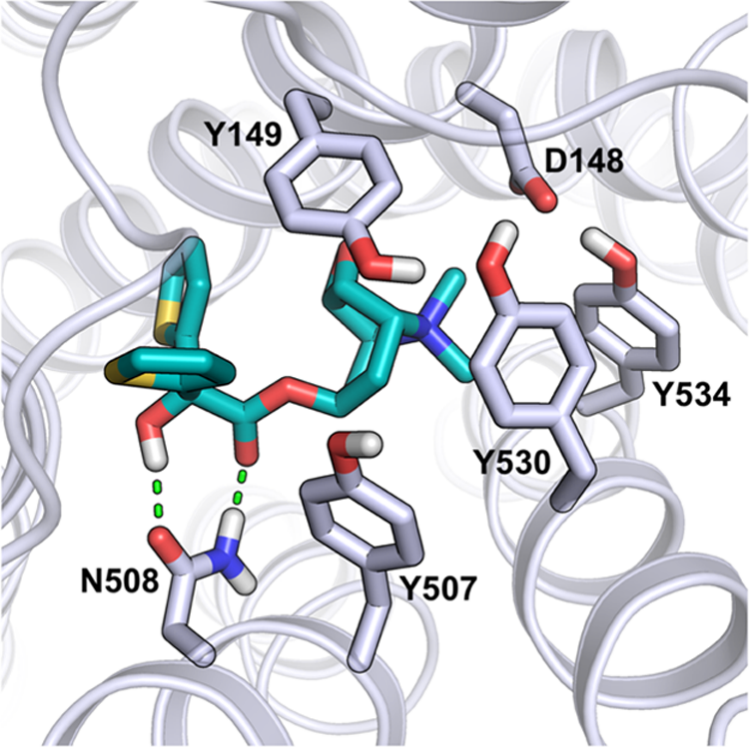
Binding mode for tiotropium (dark cyan) within the model of the *h*M3 receptor (gray). H-bonds involving Asn508^6.52^ are depicted with green dashed lines.

A three-dimensional homology model of the human M3 (*h*M3) receptor was built employing the rat X-ray structure of M3 (*r*M3) in a complex with tiotropium (PDB ID: 4U15) as a template.^42^ Subsequently, M3 antagonists reported in Figure 2 were docked within the resulting *h*M3 model structure to generate the initial set of receptor–ligand complexes. As the human and rat isoforms share 92% sequence identity, which increases to 100% within the orthosteric binding site, the resulting protein–ligand complexes were considered meaningful and useful for further atomistic investigations (RMSD between the orthosteric binding site of the X-ray structure and the homology model of 0.3 Å). Similarly to what was observed in the rat X-ray complex, visual inspection of the *h*M3-tiotropium model structure (Figures 3 and S1) showed that the ligand well occupied the orthosteric binding site of the receptor, with its α-hydroxyacetyl group forming a bidentate hydrogen bond (H-bond) interaction with the Asn508^6.52^ side chain, and with its positively charged quaternary nitrogen facing the carboxylate group of Asp148^3.32^. Besides these polar contacts, tiotropium resulted deeply buried within the transmembrane (TM) receptor core and surrounded by a cage of tyrosine residues (namely, Tyr149^3.33^, Tyr507^6.51^, Tyr530^7.39^, and Tyr534^7.43^, Ballesteros-Weinstein numbering in superscripts), sealing the orthosteric binding site and hampering the access of water molecules. This arrangement is consistent with mutagenesis data^34^ suggesting that tiotropium, after breaking the H-bond interactions with Asn508^6.52^, may need to pass through the “tyrosine cage” to exit the M3 binding site and reach the solvent bulk. The crossing of this aromatic cage has been proposed as the rate-limiting step of M3 unbinding,^43^ suggesting that the M3 receptor should follow a two-state model binding kinetics in which the ligand adopts either a receptor-bound or a receptor-unbound state, with a predominant energy barrier separating these two free-energy minima. Recent investigations have shown that interactions of tiotropium with the extracellular vestibule site of the M3 receptor may also contribute to the prolonged activity of this compound.^44,45^ This suggests that the free-energy landscape of tiotropium unbinding may be featured by two distinct free-energy barriers, the first (predominant) accounting for the tyrosine cage crossing event and the second (of minor entity) accounting for the passage through the extracellular vestibule site.^46,47^

Docking simulations with Glide software showed that all of the compounds of the Tautermann data set assumed a binding mode comparable to that of tiotropium (Figure S2). We thus wondered if a simple descriptor such as the Glide score (*G*_score_)^48,49^ could provide a quantitative model able to explain the variation in the experimental RTs measured for M3 antagonists. A not satisfying correlation between *G*_score_ values and experimental RTs was found (*r*^2^ = 0.31, Figure S3 and Table S1), indicating that approaches based on the summation of pairwise interactions collected on a static structure are too simplistic to capture free-energy differences among compounds. Conversely, atomistic simulations able to describe the intrinsic dynamic nature of the formation and breaking of electrostatic and steric interactions could be a more suitable approach for RT prediction.^50,51^ It is conceivable that the stability of M3 receptor antagonist complexes (and thus the RTs) would be likely related to the strength of the electrostatic interactions occurring between (i) Asn508^6.52^ and the acetate portion, shared by all of the compounds and (ii) Asp148^3.32^ and the positively charged *N*-alkyltropane moiety of the antagonists (Figure S2). CVs able to perturb these interactions could, in principle, be applied to describe the unbinding process.

### Setup of the Computational Protocol Based on the Conformational Flooding Algorithm

**Figure 4:**
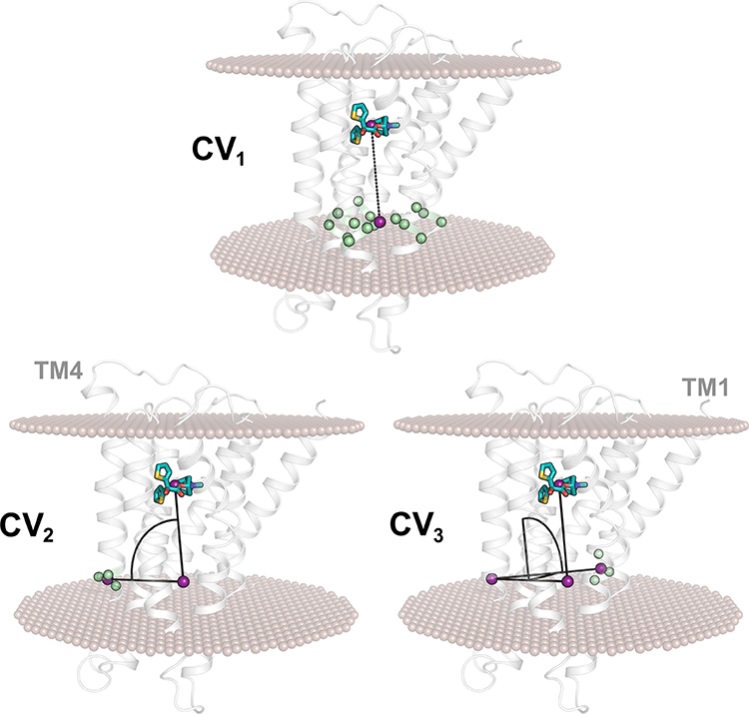
Graphical representations of the 3 CVs employed for wt-META-D simulations of tiotropium unbinding from the M3 receptor (dark cyan and gray, respectively). Key atoms employed to define the CVs are depicted with green spheres and the centers of mass (COMs) are depicted with purple spheres. See Methods for a detailed definition of CV_1_, CV_2_, and CV_3_.

We started our investigation with the setup and test of a variant of the conformational flooding method (see Methods for details). The application of this approach requires that the CVs employed to simulate the process of unbinding can unambiguously discriminate the free-energy basins corresponding to the bound and unbound states. Preliminary simulations performed with the *h*M3-tiotropium complex suggested that CVs describing translational movements of the ligand of interest could serve this scope by progressively breaking the polar interactions with the M3 binding site. We thus defined a set of three CVs able to describe the translational movements of the ligand center of mass (COM), with respect to the M3 orthosteric site, toward the solvent (Figure 4). CV_1_ functioned as a pulling variable, being defined as the distance between the COM of the antagonists and the COM of a transmembrane region of M3 close to the intracellular edge of the lipid bilayer. CV_2_ and CV_3_ described movements of the COM of the ligands along directions orthogonal to CV_1_. Specifically, CV_2_ was defined as the angle between the two COMs used for CV_1_ and a third COM localized on TM4; CV_3_ represented the dihedral angle defined by the three COMs used for CV_2_ and a fourth COM placed on TM1.

**Figure 5:**
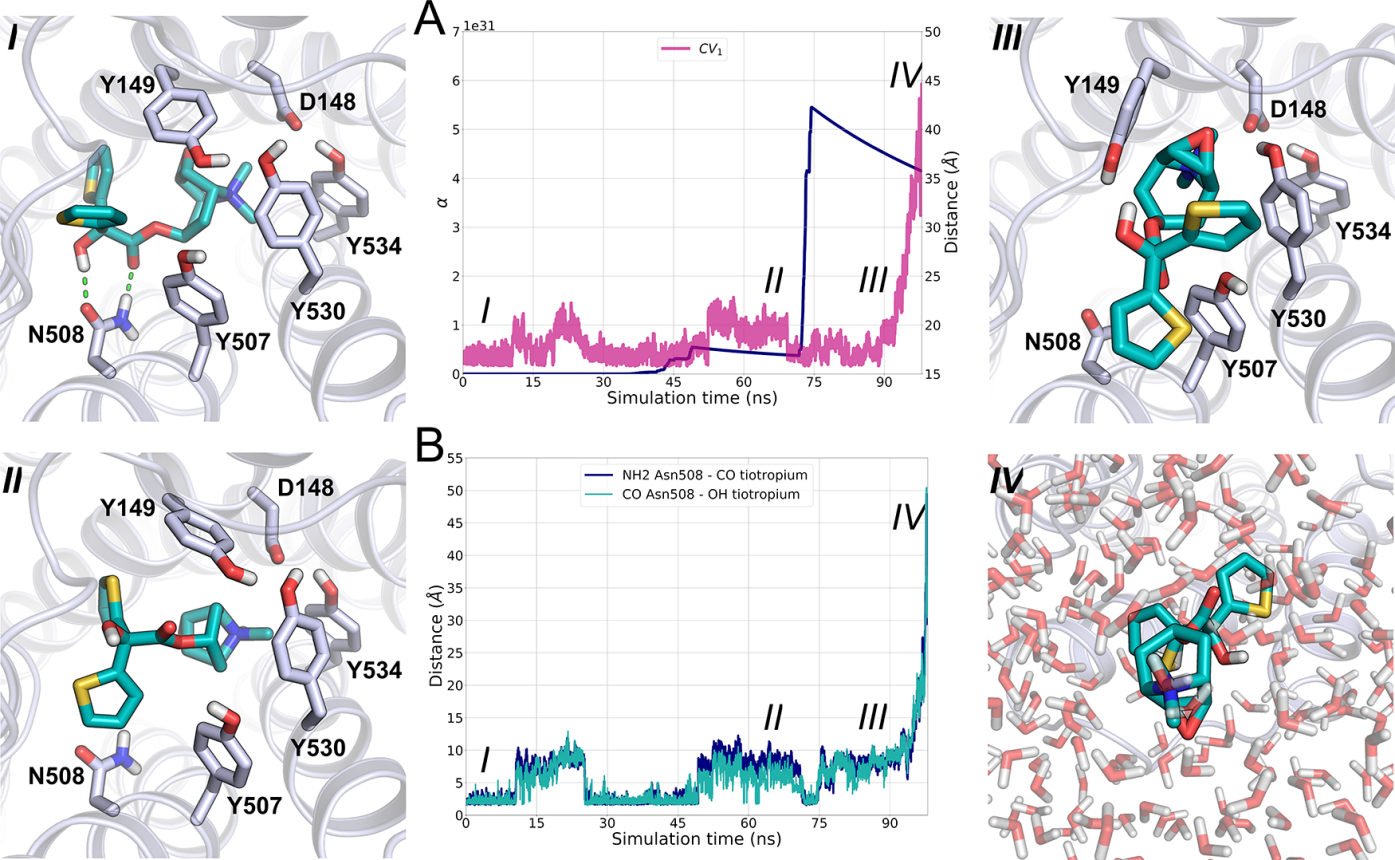
(A) Evolution of α and CV_1_ during the simulation time. (B) Evolution of the H-bonds between tiotropium and the Asn508^6.52^ side chain during the simulation time. Relevant configurations of tiotropium (dark cyan) unbinding from M3 (gray) are represented (I–IV).

Analysis of an exploratory wt-META-D simulation showed that tiotropium initially fluctuated around its initial binding pose (bound state), maintaining electrostatic interactions within the M3 orthosteric binding site for most of the simulation (Figure 5, configuration I). In this configuration, CV_1_ assumed values ranging from 15 to 20 Å (Figure 5A), while CV_2_ and CV_3_ values remained close to their starting values of nearly 90° (Figure S4). Although the increasing energy-bias deposition weakened the key H-bonds with Asn508^6.52^, the ligand was unable to cross the gate formed by tyrosine residues. As the simulation proceeded (i.e., after 75 ns of simulations), the interactions between tiotropium and the binding site became even weaker, leading to the irreversible breakage of the H-bonds involving the α–hydroxyacetate fragment of tiotropium and Asn508^6.52^ (Figure 5B, configuration II). This event was accompanied by the opening of the tyrosine cage, which allowed tiotropium to exit from the orthosteric site, passing through the extracellular vestibule and laying down on the M3 surface (Figure 5, configuration III). At this stage, CV_1_ values fluctuated around 30 Å (Figure 5A), whereas both CV_2_ and CV_3_ retained an average value of 90° (Figure S4). This configuration rapidly evolved into the unbound state (Figure 4, configuration IV), in which tiotropium was fully solvated (CV_1_ > 40 Å and CV_2_ and CV_3_ with values fluctuating around 105 and −90°, respectively; see Figures 5A and S4).

During the wt-META-D simulation, the evolution of the acceleration factor over time (see Methods for details) was also recorded to access a RT_calcd_ value for tiotropium (Figure 5A). According to the conformational flooding method, the transition from the bound to unbound state causes an abrupt change in the sign of the first derivative dα/d*t*. RT_calcd_ was then obtained from the product between the peak of α registered during the simulation and the time at which the transition occurred (i.e., the transition time). Overall, this approach seemed suitable to study M3 antagonist unbinding and thus to estimate RTs. Even in terms of computational effort, the protocol appeared affordable, as the cost of a single wt-META-D simulation was around 100 ns.

### Performance of the Conformational Flooding Protocol Based on 3 CVs

**Figure 6:**
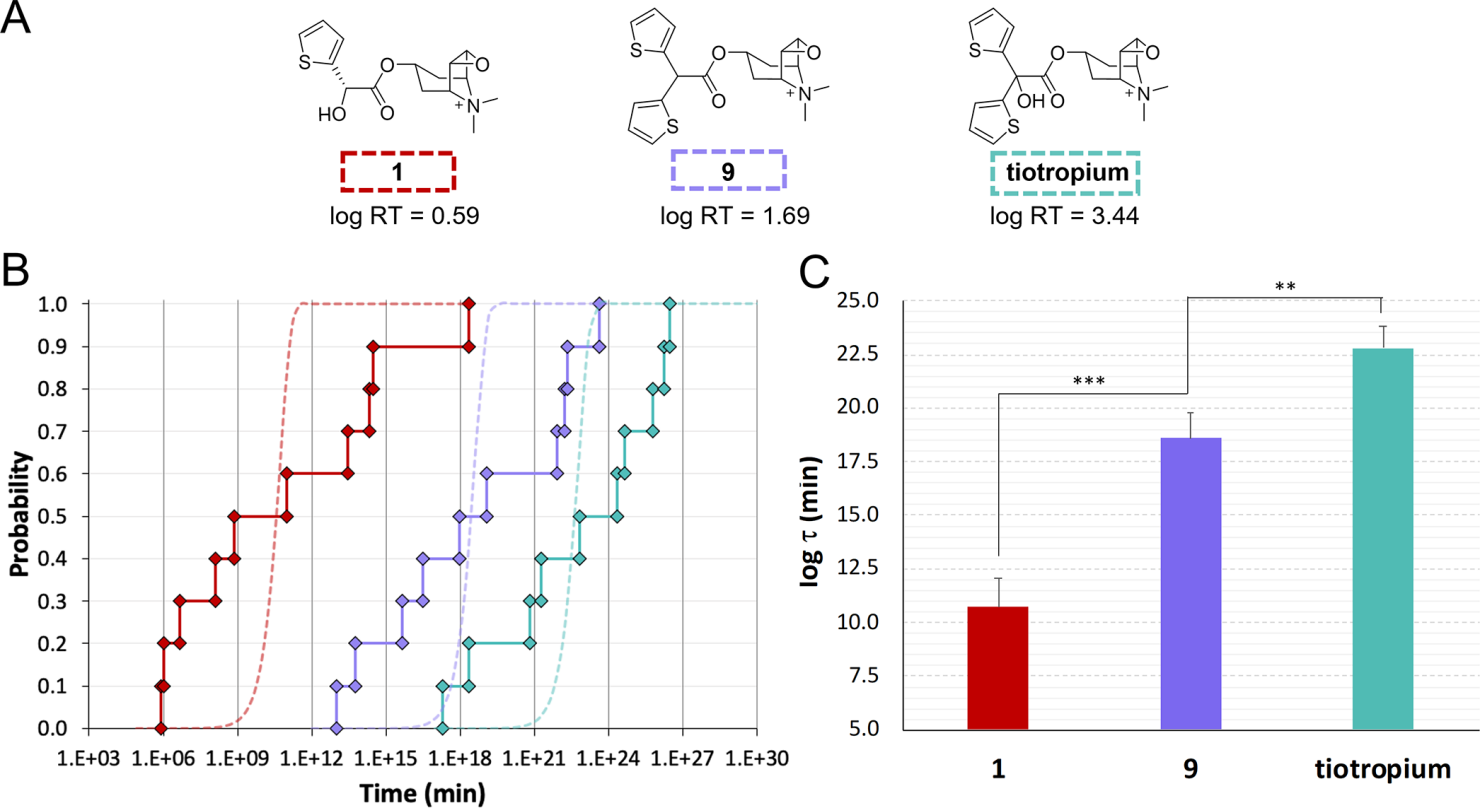
(A) Chemical structures and experimental RT values (expressed in minutes, log unit) of the M3 antagonists chosen to setup the computational protocol. (B) Empirical cumulative distributions (ECDs) of RT_calcd_ (continuous lines with diamond markers) and theoretical Poisson curves (dotted lines), described by the equation *P* = 1 – exp (−RT_calcd_/log τ), for the set of 10 independent wt-META-D of **1** (red), **9** (violet), and tiotropium (cyan) unbinding simulations. (C) Log τ expressed as the mean of log RT_calcd_ ± SEM (*n* = 10) together with the result of unpaired Student’s *t*-test (***p < 0.001, **p < 0.01).

The computational protocol reported above was applied to simulate the unbinding from the M3 receptor of a minimal set of antagonists having different experimental RTs (Figure 6A). In addition to tiotropium, featuring a long RT (LRT) (∼2724 min), **9** (also known as deoxy-tiotropium), displaying a medium RT (MRT) (∼50 min), and **1**, lacking thiophene and showing a very short RT (SRT) (∼4 min), were selected for this computational analysis.

This set was selected to assess the ability of our wt-META-D approach to predict (i) absolute RT values and (ii) the relative ranking of compounds. To evaluate the Poisson-like behavior of the unbinding process of tiotropium, **9**, and **1**, 10 independent wt-META-D simulations of unbinding were performed for each compound, and empirical cumulative distributions (ECDs) were built from collected RT_calcd_ (Figure 6B). Comparison between ECDs and theoretical cumulative distributions (described by *P* = 1 – exp (−RT_calcd_/log τ))^40^ suggested the presence of a non-Poisson process. Nevertheless, no dramatic overlaps were detected among the ECDs. To allow a meaningful comparison among distributions, the geometric mean of collected RT_calcd_ (now expressed as τ) was employed. This appeared a reasonable choice considering that log RT_calcd_ values were normally distributed and that the geometric average has already been shown to be a fair approximation of the characteristic parameter of a Poisson distribution in the context of unbinding simulations.^52^

The resulting τ values (∼10^23^ for tiotropium, ∼10^13^ for **9**, and ∼10^9^ for **1**, reported in minutes, log unit in Figure 6C), although largely overestimated with respect to the experimental RTs, correctly ranked the investigated compounds, indicating that this approach could capture differences among M3 antagonists. With the aim of extending the investigation to the entire Tautermann data set, we chose to optimize the protocol to reduce (i) RT overestimation, (ii) the uncertainty of computed values (expressed as the standard error of the mean, SEM), and (iii) the spread of ECDs.

### Optimization of the Conformational Flooding Protocol

**Figure 7:**
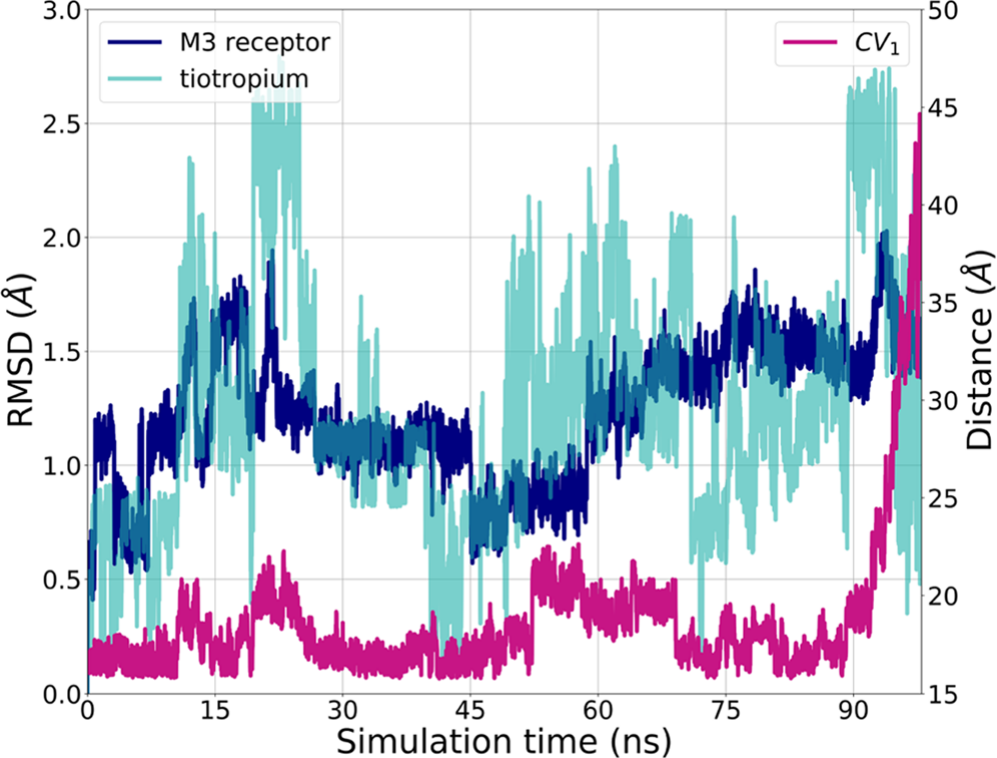
RMSD analysis of side chain residues (blue) within 5 Å of tiotropium and tiotropium heavy atoms (dark cyan) and evolution of CV_1_ (magenta) during a wt-META-D simulation of tiotropium taken as an example.

We wondered if the inclusion of additional CVs could lead to a computational approach able to provide better results in terms of accuracy and precision. Visual inspection of wt-META-D of the *h*M3-tiotropium system provided hints on slow structural rearrangements occurring during unbinding, which could require an explicit description. As anticipated before, the exit of tiotropium involved the opening of the tyrosine cage and the concurrent breakage of the H-bond network between Tyr149^3.33^ and Tyr530^7.39^. Analysis of the wt-META-D simulation trajectories with 3 CVs showed that in addition to these tyrosine residues, several side chains of the whole orthosteric binding site experienced a reversible conformational transition toward an open state of the M3 receptor (Figure 7). The same wt-META-D simulations showed that tiotropium also explored different conformational states, particularly along the bond connecting the carbonyl carbon and the aliphatic carbon bearing the two thiophenes. From these simulations, both the RMSDs of side chains delimiting the orthosteric binding site and of ligand heavy atoms (which could induce rotations around key dihedral angles) emerged as CVs accounting for relevant conformational changes occurring during the unbinding (Figures 7 and S5) and thus to be included in the conformational flooding protocol.

Two sets of 10 independent wt-META-D simulations were performed for tiotropium, **9**, and **1**, employing 4 CVs. In the first set, we added the RMSD of the side chains delimiting the orthosteric site as the 4th collective variable (4 CVs_a_), whereas, in the second one, we included the RMSD of ligand heavy atoms (4 CVs_b_). In both cases, the presence of a 4th CV led to a moderate improvement of accuracy and dispersion of RT_calcd_ values, as indicated by log τ values reported in Tables 1 and S2. Prompted by these results, we applied the conformational flooding approach to simulate the unbinding of the set of three M3 antagonists including these two new CVs (5 CV data set in Tables 1 and S2). Gathered data showed a further improvement in accuracy and uncertainty of τ values (with SEM, in general, lower than 0.7 log unit) compared to the protocol based on the 3 CVs described above. Furthermore, analysis of the unbinding trajectories for the 5 CV protocol applied to tiotropium, **9**, and **1** showed that the unbinding path was comparable to that observed with the 3 CV protocol described above.

**Table 1:** Log τ Values (Expressed in Minutes) Reported as the Mean ± SEM for Tiotropium, **9**, and **1**, Using Different Sets of CVs^a^

	3 CVs	4 CVs_a_	4 CVs_b_	5 CVs	log RT
tiotropium	22.8 ± 1.03	19.2 ± 1.02	16.9 ± 0.72	14.6 ± 0.63	3.44
**9**	18.6 ± 1.21	14.5 ± 0.85	13.0 ± 0.62	9.80 ± 0.53	1.69
**1**	10.7 ± 1.36	10.1 ± 0.85	8.02 ± 0.49	6.79 ± 0.67	0.59

a Experimental log RTs (expressed in minutes) are also reported.

**Figure 8:**
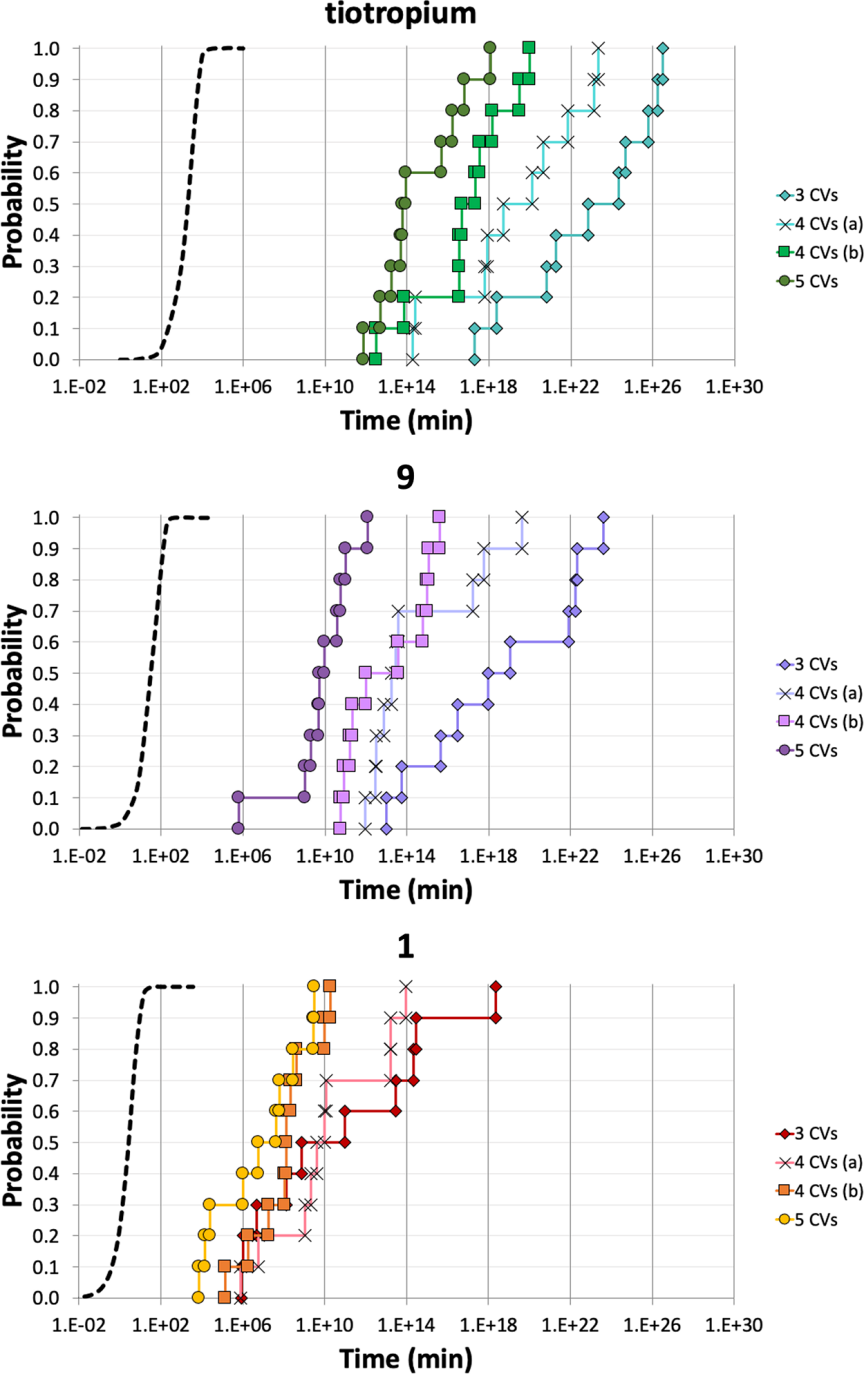
Empirical cumulative distributions (ECDs) derived from unbinding simulations for tiotropium, **9**, and **1**. Dotted black lines represent theoretical Poisson distributions with characteristic parameters corresponding to experimental RTs for tiotropium, **9**, and **1**, respectively.

The ECDs calculated for tiotropium, **9**, and **1** from RT_calcd_ values obtained with the set of 5 CVs were consistent with Poisson curves (Figure 8). This suggested that the 5 CV protocol, in which the cost of each independent wt-META-D simulation has increased up to ∼500 ns, better satisfied the requirement of applicability of the conformational flooding approach (see Methods for details). Despite this improvement, the absolute errors in the estimation of the experimental RT remained significant. Overestimation of the RT values is likely dependent on the complexity of the ligand binding/unbinding phenomena, which involve events (i.e., the major conformational transition of the receptor) or interactions of the M3 receptor itself with the key components of the biological environment (cellular membrane or proteins involved in the signal transduction) which were not considered in the virtual model.

Still, we attempted to improve the accuracy of the protocol by testing an alternative set of 5 CVs in which the translational collective variables CV_1_–CV_3_ were constructed using points located on the extracellular side of the M3 receptor (Figure S6). The ECDs calculated for tiotropium, **9**, and **1** with this new CV set, while coherent with Poisson curves, returned τ values far from experimental data, similar to what was observed for the original set of 5 CVs (Table S3 and Figure S7). Furthermore, no significant differences were observed in the unbinding path followed by the compounds applying the two sets of 5 CVs. That said, we cannot rule out that other CVs, including those based on the “path” methodology (i.e., *S* and *Z* descriptors),^53,54^ could provide a more accurate estimation of the absolute RT value.^16,55^

Considering the low uncertainty (SEM) associated with the computed log τ values and the satisfactory ability of the 5 CV protocol to discriminate M3 antagonists with long, medium, and short RT, we sought to treat log τ as an empirical descriptor and thus to search for a correlative model between it and experimental RT values, after extending the application of the protocol to the other compounds of the Tautermann data set.^34^

### Extension of the 5 CV Protocol to Other M3 Antagonists and Analysis of Its Performance

The 5 CV protocol was applied to the other M3 antagonist complexes generated by docking (i.e., based on compounds **3**, **5**–**8**, **10**, and NMS, Figure S2). Ten independent simulations were carried out for each complex and used to retrieve log τ for each compound by computing the geometric average of collected RT_calcd_ values (Table S4). In general, log τ values paralleled the experimental log RT values, suggesting that the protocol could correctly capture the effect of compound modifications on unbinding kinetics (Figures S8 and 9). Tiotropium, **6**, and **8**, displaying minor variation at the level of the tropane moiety, were predicted as long RT (LRT) antagonists in line with experimental data. Compounds **3**, **5**, **7**, and **9**, characterized by important modifications on the acetoxy group, were also correctly ranked as medium RT (MRT) antagonists, and **1**, lacking a thiophene ring, was accurately classified as a short RT (SRT) compound. Conversely, **10** and NMS, which display significant modifications in their terminal lipophilic region (both lacking thiophene rings in their structure), were somehow misclassified.

**Figure 9:**
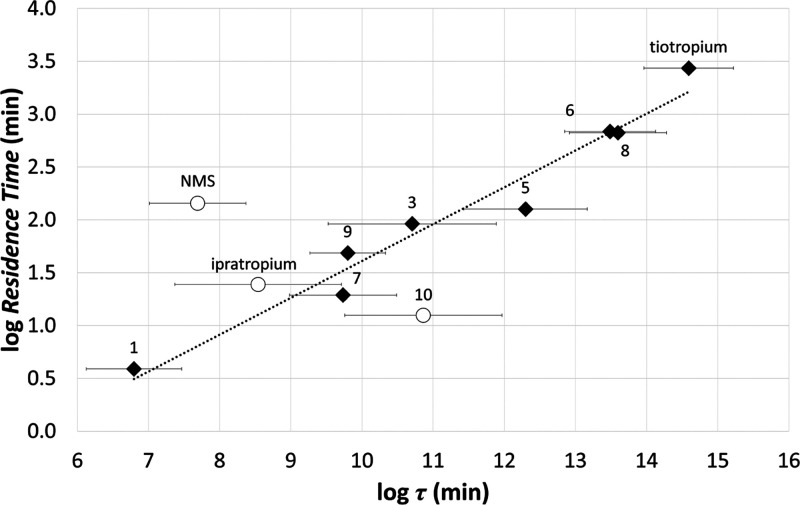
Experimental log RT values vs. computed log τ values for the Tautermann data set of M3 antagonists and for ipratropium (Glossop et al.^56^). Bars represent the SEM over 10 metadynamics replicas. The regression line was built considering only the congeneric compounds tiotropium, **1**, **3**, and **5**–**9** (black diamonds). Compounds employed as a test set (NMS, **10**, ipratropium) are also displayed (white circles).

Since **10** and NMS exhibited rather low structural similarity to tiotropium (Tanimoto similarity < 0.45) when compared to the other compounds of the Tautermann data set (Table 2), we searched for a quantitative SKR model after their exclusion. This analysis led to a quantitative SKR model (eq 1) characterized by satisfying statistical parameters, with log τ explaining 96% of log RT variation. This SKR equation resulted thus able to capture quantitative differences among M3 antagonists closely related to tiotropium with an RMSE value of only 0.20 log unit.
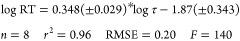
1To evaluate the impact of compound similarity on the estimation of the experimental RT, we used eq 1 to predict the RT of NMS, **10** (excluded from the regression model) and that of ipratropium, an M3 antagonist correlated to NMS (for which it is reasonable assuming a binding mode similar to NMS, see Figure S9 and Table S5) and recognized as an SRT ligand.^55^ When structural diversity increased, the absolute value of the residual *e* grew, reaching values higher than the RMSE associated with eq 1 (0.20 log unit). With the exception of NMS (*e* = 1.35), which emerged as a severe outlier, the other two compounds not included in the calibration displayed residuals lower than 1 log unit (ipratropium = 0.29; **10** = −0.82), which in the context of medicinal chemistry applications can be considered an acceptable error (Table 2).

**Table 2:** Experimental Log RT Values and Computed log τ Values (Expressed in Minutes, Log Scale) for the Tautermann Data Set

	log RT^a^	log τ^b^	log RT_pred_^c^	residual *e*^d^	similarity^e^
tiotropium	3.44	14.6 ± 0.63	3.21	0.23	1.00
**6**	2.84	13.5 ± 0.64	2.83	0.01	0.62
**8**	2.82	13.6 ± 0.69	2.86	–0.04	0.69
**5**	2.10	12.3 ± 0.87	2.41	–0.31	0.63
**3**	1.96	10.7 ± 1.18	1.85	0.11	0.63
**9**	1.69	9.80 ± 0.53	1.54	0.15	0.59
**7**	1.29	9.74 ± 0.75	1.52	–0.23	0.61
**1**	0.59	6.79 ± 0.67	0.49	0.10	0.62
NMS	2.16	7.70 ± 0.61	0.81	1.35	0.41
ipratropium^f^	1.39	8.53 ± 1.17	1.10	0.29	0.17
**10**	1.10	10.9 ± 1.10	1.92	–0.82	0.48

a Experimental log RT values.

b Computed log τ values obtained from the 5 CV wt-META-D protocol, expressed as the mean ± SEM.

c Log RT values (log RT_pred_) calculated using equation 1.

d Residual error vector *e* (calculated as log RT − log RT_pred_).

e Tanimoto similarity calculated with respect to tiotropium (see Methods).

f Data taken from Glossop et al.^56^

This analysis indicated that the optimized conformational flooding protocol described here could be used to predict the impact of chemical modification on the experimental residence time on a series of ligands binding the same target, provided that a coherent data set of experimental RT values is available for model calibration.

**Figure 10:**
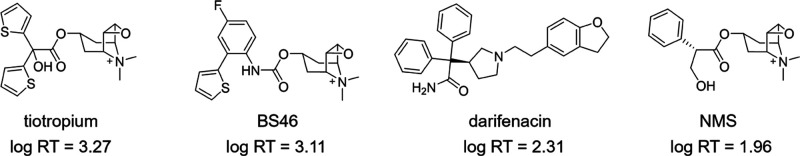
Chemical structures and experimental RT values (expressed in minutes, log unit) of tiotropium, BS46, darifenacin, and NMS, taken from the Liu data set of M3 antagonists.^56^

To further corroborate this finding, we applied the optimized conformational flooding protocol based on 5 CVs to search for a quantitative SKR for another set of M3 antagonists (Figure 10), for which the kinetic properties have been recently investigated by Liu and colleagues on *h*M3.^57^ Molecular models of BS46 and darifenacin in a complex with *h*M3 were generated by docking using the same protocol described for tiotropium and its analogues (see Methods). The identification of the binding pose was straightforward in the case of BS46 (Figure S10), as the X-ray structure of this compound in the complex *r*M3 receptor is available. In the case of darifenacin, the top-ranked pose was selected. This docking model appeared reasonable, as darifenacin exploited its carboxamide group to form H-bonds with Asn508^6.52^ and its protonated pyrrolidine ring to form a productive electrostatic interaction with Asp148^3.32^ (Figure S11).

**Figure 11:**
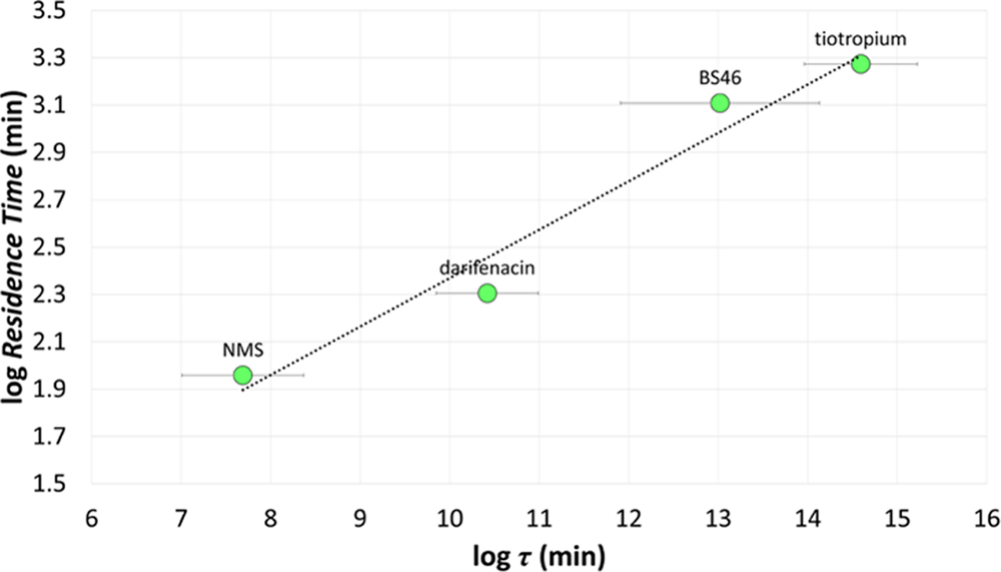
Experimental log RT vs. computed log τ values for the Liu data set of M3 antagonists^57^ displayed with green circles. Bars represent the SEM over 10 metadynamics replicas.

The resulting *h*M3-ligand complexes were equilibrated by MD simulations and submitted to the 5 CV conformational flooding protocol to retrieve the characteristic log τ parameter (Table S6). Computed log τ allowed us to correctly rank the investigated M3 antagonists according to their experimental residence time (Figure 11), with tiotropium and BS46 well separated from darifenacin and NMS.

Log τ correlated with the experimental RT providing a satisfactory SKR model represented by eq 2.
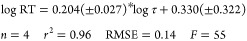
2Despite the chemical heterogeneity due to the presence of darifenacin and BS46 (Tanimoto similarity lower or equal to 0.2, see Table 3), log τ was able to explain 96% of log RT variation, with an RMSE of the model lower than a 0.20 log unit. It is worth remarking that both the regression coefficient and the intercept of eq 2 were substantially different from those obtained as the output of the regression analysis of the Tautermann congeneric series of M3 antagonists described by eq 1. This suggested that these quantitative SKRs have to be considered statistical models with local validity and that prospective estimation of the RT should be attempted once a calibration study has been performed with a set of solid kinetics data.

**Table 3:** Experimental Log RT Values and Computed log τ Values (Expressed in Minutes, Log Scale) for the Liu Data Set

	log RT^a^	log τ^b^	log RT_pred_^c^	residual *e*^d^	similarity^e^
tiotropium	3.27	14.6 ± 0.63	3.31	–0.04	1.00
BS46	3.11	13.0 ± 1.11	2.98	0.13	0.22
darifenacin	2.31	10.4 ± 0.58	2.45	–0.14	0.10
NMS	1.96	7.70 ± 0.61	1.90	0.06	0.41

a Experimental log RT values.

b Computed log τ values obtained from the 5 CV wt-META-D protocol, expressed as the mean ± SEM.

c Log RT values (log RT_pred_) calculated using equation 2.

d Residual error vector *e* (calculated as log RT − log RT_pred_).

e Tanimoto similarity calculated with respect to tiotropium (see Methods).

### Setup and Application of the Computational Protocol Based on *t*_META-D_ to M3 Antagonists

To further investigate and test the ability of META-D-based protocols to provide a ranking for M3 antagonists in agreement with experimental RTs, we applied the empirical protocol *t*_META-D_,^41^ which is simply based on the time of the META-D simulation necessary to observe ligand unbinding. In this approach, which does not require well-tempering conditions, the total potential deposited over the CV space is proportional to the simulation time spent by the compound in the bound state (i.e., the *t*_META-D_). In our case, the simulation was considered ended, and the *t*_META-D_ value was computed when the system reached the unbound state, a condition that was satisfied when the ligand was fully solvated by water molecules without protein residues within a sphere of a 5 Å radius from any of its atoms.

The set of translational CVs (CV_1_–CV_3_) initially defined for the conformational flooding protocol was also employed in the case of the *t*_META-D_ protocol in light of their simplicity, generally in the context of GPCR systems, and ability to be effective in describing the unbinding of tiotropium, **9**, and **1** from the *h*M3 receptor. To account for statistical uncertainty, *t*_META-D_ values were collected from 10 independent replicas for each ligand. The resulting average value was used as an energetic descriptor to search for qualitative and/or quantitative models to describe variation in experimental RT values (Table S7). We anticipate that, differently from the conformational flooding approach, unbinding of M3 antagonists with the *t*_META-D_ protocol occurred in less than 50 ns of simulation, making this approach computationally affordable.

In general, *t*_META-D_ values well paralleled experimental data (Figures S12 and 12), showing LRT antagonists (tiotropium, **6**, **8**) well separated from SRT antagonists (**1**, **7**, **10**). The behavior of MRT antagonists (**3**, **5**, **9**, and NMS) resulted in being somehow erratic, particularly in the case of **3** and NMS.

**Figure 12:**
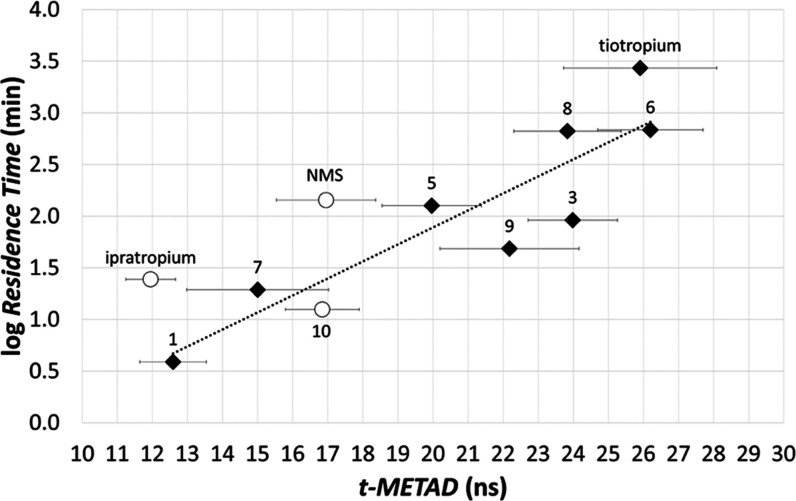
Experimental log RT values vs. computed *t*_META-D_ values for the Tautermann data set and for ipratropium (Glossop et al.^55^). Bars represent the SEM over 10 metadynamics replicas. The regression line was built by considering congeneric compounds (i.e., tiotropium, **1**, **3**, and **5**–**9**).

Similar to what was performed in the case of the conformational flooding algorithm, we searched for a quantitative SKR model after the exclusion of NMS and **10** from the regression analysis. The resulting equation (eq 3) gave fair statistical parameters with the *t*_META-D_ descriptor able to account for the 80% variation in the experimental log RT. The RMSE value accompanying eq 3 (0.45) was reasonable but slightly higher than that obtained with the SKR model based on log τ.
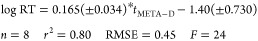
3Equation 3 was next applied to estimate the RT of NMS, **10** (excluded from the regression analysis), and ipratropium. While compound **10** was well described by the model (*e* = −0.28, Table 4), both NMS and ipratropium displayed a rather high residual (∼0.80 log unit, Tables 4 and S8), as they were predicted to dissociate from the M3 receptor faster than expected. Different to what was observed with the conformational flooding algorithm, in which the SKR model based on the log τ parameter produced an RT estimation for NMS with a residual of 1.35 log unit, in the case of the *t*_META-D_-based SKR, there was no evidence of severe outliers. This finding suggested that the *t*_META-D_ descriptor may be less sensitive to structural diversity than log τ obtained through the conformational flooding approach.

**Table 4:** Experimental Log RT Values and Computed *t*_META-D_ Values (Expressed in Minutes and Nanoseconds, Respectively) for the Tautermann Data Set

	log RT^a^	*t*_META-D_^b^	log RT_pred_^c^	residual *e*^d^	similarity^e^
tiotropium	3.44	25.9 ± 2.19	2.86	0.58	1.00
**6**	2.84	26.2 ± 1.50	2.91	–0.07	0.62
**8**	2.82	23.8 ± 1.53	2.52	0.30	0.69
**5**	2.10	20.0 ± 1.27	1.89	0.21	0.63
**3**	1.96	24.0 ± 1.98	2.55	–0.59	0.63
**9**	1.69	22.2 ± 2.02	2.25	–0.56	0.59
**7**	1.29	15.0 ± 0.95	1.07	0.22	0.61
**1**	0.59	12.6 ± 0.70	0.67	–0.08	0.62
NMS	2.16	17.0 ± 1.42	1.40	0.76	0.41
ipratropium^f^	1.39	12.0 ± 0.71	0.57	0.82	0.17
**10**	1.10	16.9 ± 1.05	1.38	–0.28	0.48

a Experimental log RT values.

b Computed *t*_META-D_ values obtained from the 3 CV META-D protocol, expressed as the mean ± SEM.

c Log RT values (log RT_pred_) calculated using equation 3.

d Residual error vector *e* (calculated as log RT − log RT_pred_).

e Tanimoto similarity calculated with respect to tiotropium (see Methods).

f Data taken from Glossop et al.^56^

**Figure 13:**
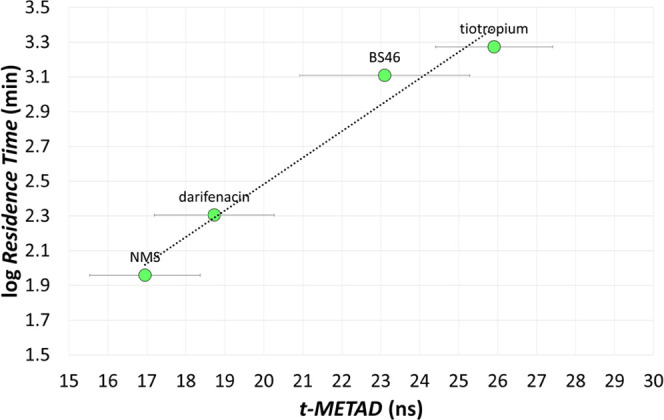
Experimental log RT vs. computed *t*_META-D_ values for the Liu data set of M3 antagonists displayed with green circles. Bars represent the SEM over 10 metadynamics replicas.

As a final step of our investigation, we applied the *t*_META-D_ approach to model the RT parameter of four M3 antagonists described by Liu et al. (Table S9).^56^ This analysis, represented in Figure 13, showed that also *t*_META-D_ provided a fair ranking of these M3 antagonists.

In addition, the *t*_META-D_ descriptor correlated with the experimental RT values providing an SKR model, represented by eq 4, with statistical parameters and residual *e* (Table 5) in line with those accompanying the regression model based on log τ as a descriptor (eq 2):
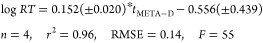
4SKR equations derived from the *t*_META-D_ descriptor (i.e., eqs 3 and 4) displayed values of both the regression coefficient and the intercept, which were data set dependent, confirming their local validity. A prospective estimation of RT should be performed after calibration with a set of homogenous kinetics data also in the case of the *t*_META-D_ protocol.

**Table 5:** Experimental Log RT Values and Computed *t*_META-D_ Values (Expressed in Minutes and Nanoseconds, Respectively) for the Liu Data Set

	log RT^a^	*t*_META-D_^b^	log RT_pred_^c^	residual *e*^d^	similarity^e^
tiotropium	3.27	25.9 ± 2.19	3.38	–0.11	1.00
BS46	3.11	23.1 ± 1.12	2.96	0.15	0.22
darifenacin	2.31	18.7 ± 1.68	2.29	0.02	0.10
NMS	1.96	17.0 ± 1.42	2.03	–0.07	0.41

a Experimental log RT values.

b Computed *t*_META-D_ values obtained from the 3 CV META-D protocol, expressed as the mean ± SEM.

c Log RT values (log RT_pred_) calculated using equation 4.

d Residual error vector *e* (calculated as log RT − log RT_pred_).

e Tanimoto similarity calculated with respect to tiotropium (see Methods).

## Discussion

Computational efforts in the context of RT prediction have led to the development of several approaches that could be effectively used in the context of SKR investigation and lead RT optimization at an affordable computational cost.^13,58^ Within this framework, two META-D-based protocols were here applied to search for quantitative models able to predict the experimental RTs for a set of M3 antagonists derived from tiotropium.

We started our investigation by setting up a computational protocol based on the conformational flooding algorithm. This protocol was preliminarily applied on a set of three representative M3 antagonists endowed with long (tiotropium), medium (**9**), and short RT (**1**). Although the protocol was able to distinguish the compounds into three classes, the range of computed log τ was too narrow to attempt the ranking of all of the other compounds of the data set. A rational optimization of the parameters allowed us to identify a protocol based on 5 CVs, which encoded conformational rearrangements of both the protein and ligand during unbinding. This set of parameters was able to broaden the range between the three reference compounds, thus allowing an effective ranking of a whole set of M3 antagonists considered in the study. The application of the 5 CV protocol to a subset of highly similar M3 antagonists allowed us to obtain a significant quantitative SKR model, with an RMSE of a 0.20 log unit, suggesting that a quantitative estimation could be achieved in the presence of homogeneous and consistent experimental data.

We also evaluated the ability of the conformational flooding algorithm to correctly rank a second set of compounds, composed of tiotropium, NMS, and two M3 antagonists belonging to distinct chemical classes, i.e., carboxamide darifenacin and carbamate BS46. The results of this additional investigation showed that computed log τ values were able to reproduce the experimental ranking, with NMS being well described by the model. It is fundamental to remark that regression coefficients that accompany quantitative SKRs are system-dependent, indicating that estimation of experimental RT could be attempted once a calibration study has been performed. When this condition is satisfied, this approach can be used to predict the impact of a chemical modification on the kinetics of unbinding, thus assisting ligand optimization.

Given the computational cost of this approach, we also evaluated the application of a protocol based on the *t*_META-D_ parameter. The *t*_META-D_ approach was able to rank compounds according to experimental RTs, although with an estimation of the experimental RT affected by higher uncertainty compared to the conformational flooding algorithm.

## Conclusions

Our work confirmed the ability of enhanced sampling simulations to capture differences among ligands possessing different RTs, indicating that META-D protocols can be effectively applied to help the elucidation of SKR, at least in the area of muscarinic receptor antagonists. The conformational flooding approach (with τ employed as an empirical descriptor) emerged as a fair approach to attempt quantitative prediction. The *t*_META-D_ algorithm displayed a certain ability to discriminate ligands featuring short, medium, and long residence time, although with higher uncertainty in the quantitative estimation of the RT. Overall, both computational protocols could be used to predict the impact of a chemical modification on the experimental residence time of a given ligand once a calibration with a subset of compounds has been performed.

## Methods

### Model Building

The crystal structure of tiotropium in a complex with the rat homologue of the M3 (*r*M3) receptor was downloaded from the Protein Data Bank (PDB ID: 4U15),^42^ and its chain A was used as a template structure to build the homology model of tiotropium in a complex with the *h*M3 receptor. The amino acid sequence of the *h*M3 receptor was obtained from the Universal Protein Resource^59^ (UniProt ID: P20309), and it shares an identity of 92% with the rat sequence (UniProt ID: P08483). In the *r*M3 X-ray structure, the intracellular loop 3 (ICL3) is replaced by T4-lysozyme (T4L). Thus, before building the homology model of *h*M3, the portion corresponding to T4L was manually removed from the template structure (residues from M1001 to T1118 of PDB ID: 4U15, chain A). Since the ICL3 domain of the *h*M3 receptor (residues from R261 to S482 of Uniprot ID: P20309) had no reliable template for modeling, it was not included in the final model structure. Sequence alignment between the template X-ray structure of the *r*M3 receptor and the target *h*M3 receptor was performed with Prime.^60^ The final sequence alignment is reported in Figure S13, revealing a sequence identity of 95%. Finally, six amino acids (alignment positions of 65, 77, 92, 146, 525, and 557 of the *h*M3 receptor) were mutated by the knowledge-based builder of Prime.^59^ The Ramachandran plot of the *h*M3 homology model showed that most of the residues were located in the dihedral angle zones, allowing for protein secondary structures (Figure S14), indicating that the comparative modeling process generated a geometry consistent with the crystal used as a template. The final model of the *h*M3 receptor was then processed using the Protein Preparation Wizard.^61^ Hydrogen atoms were added, and the generated hydrogen bonding networks were optimized by sampling the orientation of hydroxyl and thiol groups, together with the side chain amides of asparagine and glutamine residues. The same procedure was performed for NMS by exploiting the X-ray structure in which it is in a complex with the *r*M3 receptor (PDB ID: 4U16).^42^ All of the other M3 antagonists from the Tautermann data set were modeled with Maestro and then docked within the prepared model of the *h*M3 receptor (built from the X-ray of *r*M3 in a complex with tiotropium) using Glide.^47,48^ For each antagonist, the pose showing the lowest RMSD from the crystal pose of tiotropium in a complex with the *r*M3 receptor was retained. The same strategy was applied to the carbamate BS46, for which the X-ray structure in a complex with *r*M3 is available. For ipratropium and darifenacin, the best-ranked pose, according to *G*_score_, was selected.

Finally, in all complexes, hydrogen atoms, protein side chains, and the ligand were energy-minimized with Macromodel.^62^ The final *h*M3 antagonist complexes were embedded in a POPC lipid bilayer, solvated by SPC water molecules in a simulation box 10 Å distant from the protein in every direction, and neutralized by the addition of 15 Cl^–^ ions. The membrane was relaxed, and the system was equilibrated for 1 ns of MD simulations (500 ps in the NPT ensemble and 500 ps in the NVT ensemble) at 300 K using the Langevin thermostat.^63^ Harmonic restraints of 0.6 kcal·mol^–1^·Å^–2^ were applied on backbone heavy atoms. All bond lengths to hydrogen atoms were constrained using M-SHAKE. Short-range electrostatic interactions were cut off at 9 Å, whereas long-range electrostatic interactions were computed using the particle mesh Ewald method.^64^ A RESPA integrator was used with a time step of 2 fs, and long-range electrostatics were computed every 6 fs. MD simulations were performed using the OPLS4 force field^65^ in Desmond.^66^

### wt-META-D Simulations for Unbinding Kinetics

Equilibrated complexes were submitted to wt-META-D simulations, which allow us to enhance protein conformational changes^67,68^ and to investigate protein–ligand binding processes,^69,70^ using the Desmond 6.6 software^65^ (GPU implementation) on NVDIA GeForce RTX 3070 graphic cards. To this end, after some preliminary tests, we identified a set of 5 CVs to simulate the unbinding process of the antagonists from the M3 receptor. CV_1_ defined the distance between the COM of tiotropium heavy atoms and the COM of 15 atoms of TM2, TM3, TM5, TM6, and TM7 (three α carbons close to the membrane were chosen on each helix). CV_2_ represented the angle defined by the two COMs defined for CV_1_ and the COM of three α carbons of TM4 close to the membrane. CV_3_ defined the dihedral angle defined by the three COMs used for CV_2_ and the COM of three α carbons of TM1 close to the membrane. CV_4_ was the RMSD of side chain residues within 5 Å of tiotropium and tiotropium heavy atoms and CV_5_ represented the RMSD of ligand heavy atoms. Gaussians were deposited every 1 ps to obtain the full unbinding of the ligand. A Gaussian height of 0.40 kcal mol^–1^ was used. The width of the Gaussians was set to 0.2 Å on the distance CV_1_, 1.5° on the angle CV_2_, 1.5° on the torsion angle CV_3_, 0.1 Å on the ligand RMSD CV_4_, and 0.2 Å on the protein orthosteric binding site RMSD CV_5_. In wt-META-D, the Gaussian heights w_j_ were resized, taking into account the value of the accumulated bias potential V(*s*, *t*):
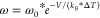
5where ω_0_ is the initial hill height, *V* is the bias potential, *k*_B_ is the Boltzmann constant, and Δ*T* is the sampling temperature, which was set to a value of around 5000 K. The product of *k*_B_ × Δ*T* corresponds to the bias factor (*kT*). In this work, the *kT* value was set to 10.

Other specific metadynamics parameters were applied. In detail, on CV_1_, a floor of 15 Å was added to desist the ligand from an erratic exit toward the intracellular portion, and on CV_5_, a wall of 3 Å was set to avoid huge and unphysical conformational changes of the *h*M3 binding site. Harmonic restraints of 0.6 kcal·mol^–1^·Å^–2^ were applied on backbone heavy atoms. All wt-META-D simulations were stopped when the ligand resulted in being unbound from the *h*M3 receptor and completely solvated. With this aim, the ligand was considered unbound from *h*M3 when the number of *h*M3 receptor atoms within 5 Å of any ligand atom was equal to zero.

### Conformational Flooding Approach

According to the conformational flooding method developed by Tiwary and Parinello,^37^ RT estimation through the application of the conformational flooding method requires that some critical conditions are satisfied: (i) CVs employed to drive the system out of the starting basin should be able to clearly distinguish the two basins; (ii) no biasing potential should be deposited in the transition state area to avoid the exploration of unphysical regions of the free-energy landscape; and (iii) the free-energy barrier in between the two basins should be larger than RT and impede spontaneous transitions.

To approach the first prerequisite, we selected CVs able to drive the system from the bound to the unbound state and accounting for orthogonal movements. For the second requirement, the time interval between two successive depositions of the biasing was set to 1 ps. To fulfill the third condition, we checked that no spontaneous ligand unbinding was observed after tens of nanoseconds in a classical MD simulation for the three reference compounds.

Assuming that the unbinding process of M3 antagonists can be simplified to a two-state model,^37^ in which the ligand can be found in its protein-bound (A) or protein-unbound state (B) with a dominant free-energy barrier separating these two basins, the RT of a simulation can be estimated as the product of the acceleration factor α and the transition time *t*, as reported in the following equation (eq 6):
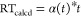
6Herein, α represents the integral over time of the potential deposited on the bound basin A to prompt the transition of the ligand from free-energy basin A to basin B and can be calculated as the running average of the bias potential accumulated through the course of a wt-META-D simulation.
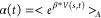
7where the angle brackets with the subscript “*A*” denote an average over a wt-META-D run confined to free-energy basin *A*, β is the Boltzmann factor, *V*(*s*, *t*) is the metadynamics bias, and *s* represents the collective variable being biased.

The transition time *t* is the time at which the ligand moves from the bound to the unbound configuration and corresponds to the simulation time at which the maximum value of α is observed, i.e., when an abrupt change in the sign of the first derivative of α with respect to the simulation time is registered.

In principle, when the required points are met, the calculated RTs for different simulations conducted on the same molecular process should adopt a Poisson distribution in agreement with the law of rare events. To account for stochastic differences among independent simulations, transition times from several replicas needed to be collected, and their distribution was interpolated by a Poisson equation to get a characteristic parameter. Alternatively, the simple application of the geometric average can return a meaningful representative parameter able to capture the information encoded in several simulations, as shown in ref (51).

With this in mind, we calculated α for each wt-META-D simulation of *h*M3 in a complex with the antagonists under evaluation, and we registered the maximum value of α, corresponding to the crossing of the highest energy barrier before reaching the unbound state and the simulation time *t* at which this value was reached. Then, the calculated residence time RT_calcd_ for each ligand at the *h*M3 receptor was estimated for each replica of the wt-META-D simulation by applying eq 6. 10 wt-META-D replicas were performed with different seeds to assign the initial atom velocities. Considering the non-normal distribution of RT_calcd_, we calculated the geometric mean (τ) as the representative parameter of the distribution for each antagonist unbinding and we reported the RT value for each compound as the mean ± SEM (*n* = 10).

### META-D Simulations for Unbinding Kinetics

Equilibrated *h*M3 antagonist complexes were submitted to META-D simulations using the same hardware/software reported above for wt-META-D. The first three CVs (CV_1_, CV_2_, and CV_3_) were used to simulate the unbinding of the antagonists from the *h*M3 orthosteric site. Gaussians were deposited every 1 ps to obtain the full unbinding of the ligand. A Gaussian height of 0.25 kcal mol^–1^ was used and no well-tempering conditions were applied. The width of the Gaussians was set to 0.2 Å on the distance CV_1_, 1.5° on the angle CV_2_, and 1.5° on the torsion angle CV_3_. On CV_1_, a floor of a 15 Å function was also added to avoid the ligand from exiting toward the intracellular portion. Harmonic restraints of 0.6 kcal·mol^–1^·Å^–2^ were applied on backbone heavy atoms. The simulations were stopped when the ligand resulted in being unbound from the *h*M3 receptor and completely solvated.

### *t*_META-D_ Approach

To measure the parameter *t*_META-D_, which corresponds to the total amount of the deposited potential required to trigger the ligand from the bound to the unbound state and accounts for all of the free-energy barriers separating bound and unbound basins, we recorded the simulation time at which the ligand is in the unbound state, considering the number of *h*M3 receptor atoms within 5 Å of any ligand atom equal to zero as exit criterion. 10 META-D replicas were performed with different seeds to assign the initial atom velocities, and the final *t*_META-D_ value for each compound was reported as the mean ± SEM (*n* = 10).

### Regression Analysis

Linear regression analyses were performed with an Excel (Microsoft Co., version 16.68) spreadsheet, employing the built-in statistical functions and automated macro procedures. Experimental log RTs and the computed log τ/*t*_META-D_ values of each compound were used as Y and X vectors, respectively. Most of the experimental log RTs of the compounds under investigation come from the Tautermann data set,^34^ with the exception of ipratropium, BS46, and darifenacin, for which the experimental RTs were retrieved from the half-life dissociation time values.^55,56^

### Tanimoto Similarity with Fingerprints

The Tanimoto similarity was computed with the Fingerprint Similarity tool implemented in Schrödinger,^71−73^ using the tiotropium structure as reference one. Radial fingerprints, also known as extended connectivity fingerprints (ECFPs), in which fragments of the structure grow radially from each heavy atom, were employed. With radial fingerprints, a specific integer value is associated with each chemically unique fragment by hashing a description of the atoms and the bonds of the fragment itself and the bonds that link it to other fragments.

## Data Availability

Molecular structures, input files used to run the simulations, and in-house Python, tcl, and bash scripts used to compute the values of log RT_calcd_ and *t*_META-D_ are made available in the Supporting Information. Schrödinger Suite 2021 (https://www.schrodinger.com) is distributed under license.

## Supporting Information Available

Binding modes for the compounds under investigation, correlation between *G*_score_ values and RTs, evolution of CV_2_ and CV_3_ during an unbinding simulation of tiotropium from the M3 receptor, definition of CV_4_ and CV_5_, computational results obtained by the application of the conformational flooding approach and of the *t*_META-D_ protocol, details on homology modeling results (PDF)
Binding modes for the compounds under investigation (ZIP)
Input files for metadynamics simulations with Desmond 6.6 implemented in Schrödinger for both the protocols applied (ZIP)
In-house scripts for the calculation of log RT_calcd_ (ZIP)
In-house scripts for the calculation of *t*_META-D_ (ZIP)

## Abbreviations Used

RT: residence time
M3: muscarinic type 3
SKR: structure–kinetics relationship
META-D: metadynamics
wt-META-D: well-tempered metadynamics
CV: collective variable
RMSE: root mean square error
ECD: empirical cumulative distribution
