# Supplementary material for: Unbinding Kinetics of Muscarinic M3 Receptor Antagonists Explained by Metadynamics Simulations
Source: J Chem Inf Model. 2023 Apr 13;63(9):2842–56. doi: 10.1021/acs.jcim.3c00042 (PMC10170513; doi:10.1021/acs.jcim.3c00042)
Supplement: Supplementary file 1 — ci3c00042_si_001.pdf [file ci3c00042_si_001.pdf]

Supporting Information for

Unbinding Kinetics of Muscarinic M3 receptor Antagonists explained by Metadynamics Simulations

Francesca Galvani, [1] *Daniele Pala,* [2] *Alberto Cuzzolin,* [2] *Laura Scalvini,* [1] *Alessio Lodola,* [1*]
Marco Mor[1,3] *and Andrea Rizzi*[2]

[1]Dipartimento di Scienze degli Alimenti e del Farmaco, Università degli Studi di Parma, Parco Area delle Scienze 27/A, I-43124 Parma, Italy

[2]Chemistry Research and Drug Design Department, Chiesi Farmaceutici S.p.A., Largo F. Belloli 11/A, 43122 Parma, Italy

[3]Microbiome Research Hub, University of Parma, Parco Area delle Scienze 11/A, I-43124 Parma, Italy

AUTHOR INFORMATION

Corresponding Author

* Phone: +39 0521 905062. Fax: + 39 0521 905006. E-mail: alessio.lodola@unipr.it

TABLE OF CONTENTS

Homology model of *h*M3 in complex with tiotropium. 3
Binding modes for the dataset of M3 antagonists. 4
G_{score} implemented by Glide as descriptor for the ranking of M3 antagonists. 5
Evolution of CV_2 and CV_3 during tiotropium unbinding simulation. 7
Definition of the CV_4 and CV_5. 7
Feasibility and optimization of the *conformational flooding* protocol. 8
Extension of the *conformational flooding* protocol with 5 CVs to the entire dataset. 12
Binding mode of ipratropium into M3 receptor. 13
Extension of the *conformational flooding* protocol with 5 CVs to ipratropium. 14
Binding mode of BS46 and darifenacin into M3. 15
Extension of the *conformational flooding* protocol with 5 CVs to BS46 and darifenacin. 16
Application of the $t_{META\text{-}D}$ protocol with 3 CVs to the entire dataset. 17
Extension of the $t_{META\text{-}D}$ protocol with 3 CVs to ipratropium. 19
Extension of the $t_{META\text{-}D}$ protocol with 3 CVs to BS46 and darifenacin. 20
Details on the homology modelling procedure for *h*M3. 21
References 22

Homology model of *h*M3 in complex with tiotropium.

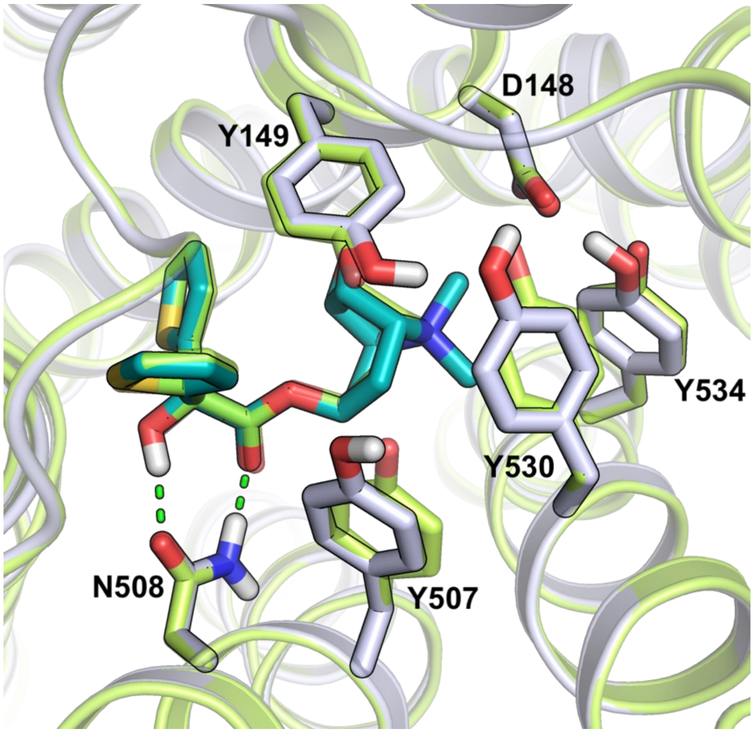

Figure S1. Molecular model of *h*M3 (gray) in complex with tiotropium (dark cyan), superimposed to the X-ray structure of *r*M3 in complex with tiotropium (light green), used as template structure. Hydrogen bonds involving tiotropium and Asn508$^{6.52}$ are depicted with green dashed lines.

Binding modes for the dataset of M3 antagonists.

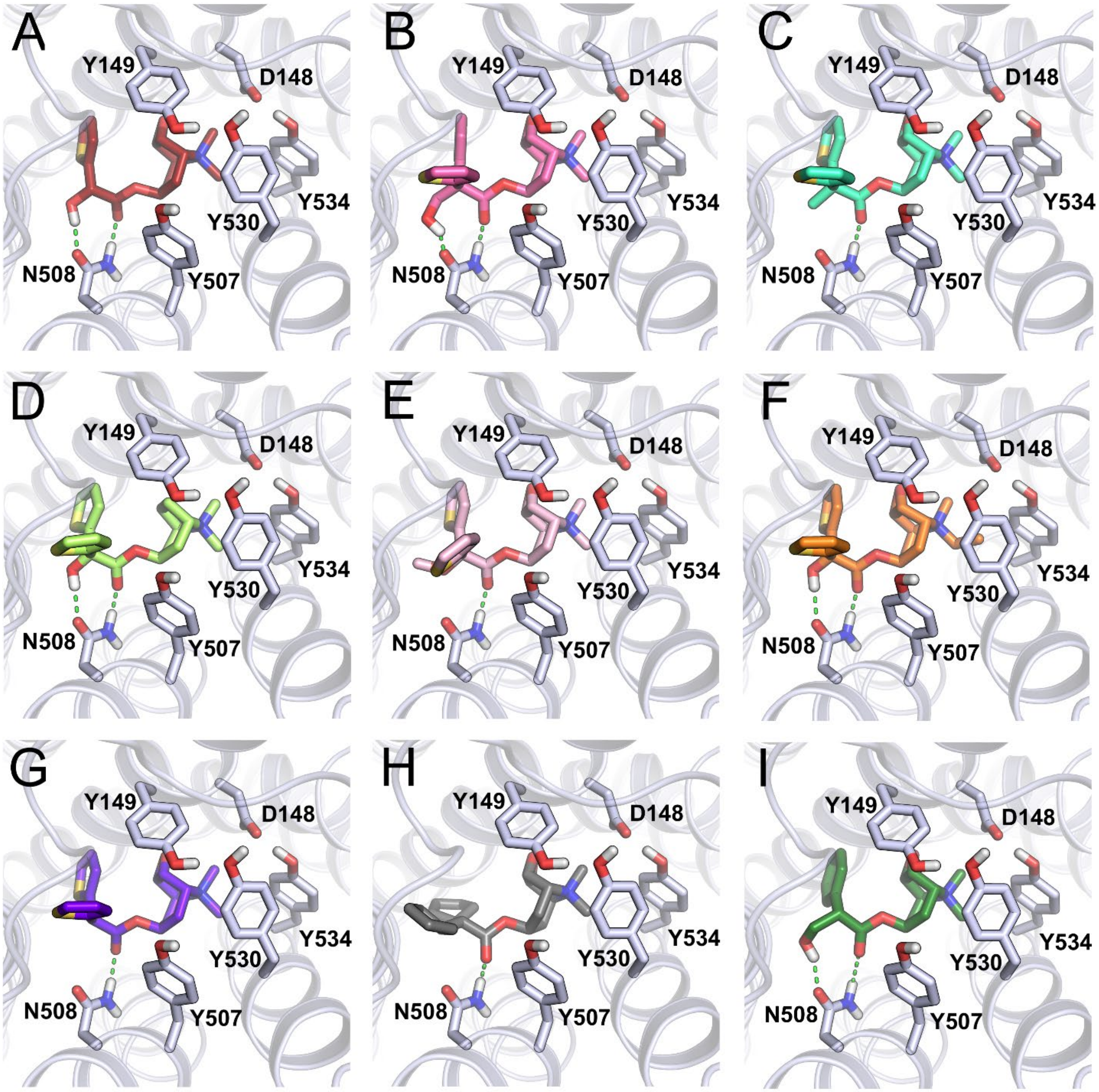

Figure S2. Molecular models of **1** (A), **3** (B), **5**-**10** (C-H) and NMS (I) within *h*M3 orthosteric binding site (gray). Hydrogen bonds involving the ligands and Asn508$^{6.52}$ are depicted with green dashed lines.

G_{score} implemented by Glide as descriptor for the ranking of M3 antagonists.

Eq. S1 describes the moderate correlation between G_{score} values obtained by docking with OPLS4 force field (Schrödinger)[1],[2] and the experimental RTs (expressed as log unit) for the Tautermann dataset of M3 antagonists.[3]

$$\log RT = -0.509\ (\pm 0.267) * \log G_{SCORE} - 3.46\ (\pm 2.87) \qquad \text{(eq. S1)}$$

$$n = 10 \quad r^2 = 0.31 \quad RMSE = 0.77 \quad F = 3.6$$

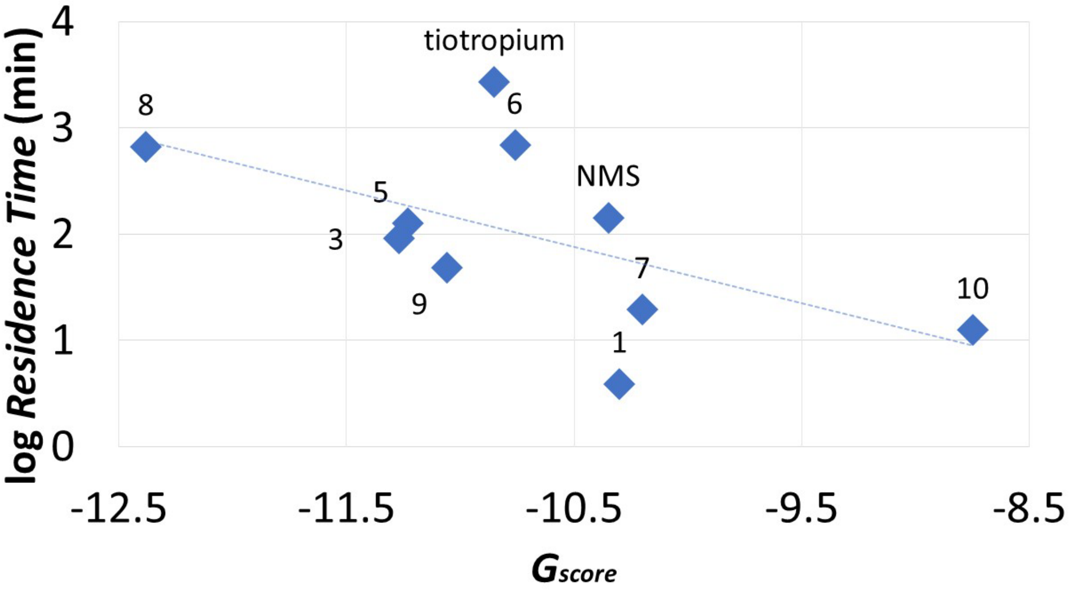

Figure S3. Plot of the experimentally observed log RT *vs* G_{score} values for the whole dataset of M3 antagonist.

Table S1. Experimental log RT values from the Tautermann dataset,[3] G_{score} values obtained by docking with OPLS4 force field (Schrödinger),[1,2] and log RT_{pred} calculated by applying equation S1 along with residual error *e* (calculated as log RT - log RT_{pred}).

	**log RT**	**G_{score}**	**log RT_{pred}**	**Residual *e***
**tiotropium**	3.44	-10.8	2.03	1.41
**6**	2.84	-10.8	2.03	0.81
**8**	2.82	-12.5	2.90	-0.08
**NMS**	2.16	-10.4	1.83	0.33
**5**	2.10	-11.2	2.24	-0.14
**3**	1.96	-11.3	2.29	-0.33
**9**	1.69	-11.1	2.18	-0.49
**7**	1.29	-10.2	1.73	-0.44
**10**	1.10	-8.75	0.99	0.11
**1**	0.59	-10.3	1.78	-1.19

Evolution of CV_2 and CV_3 during tiotropium unbinding simulation.

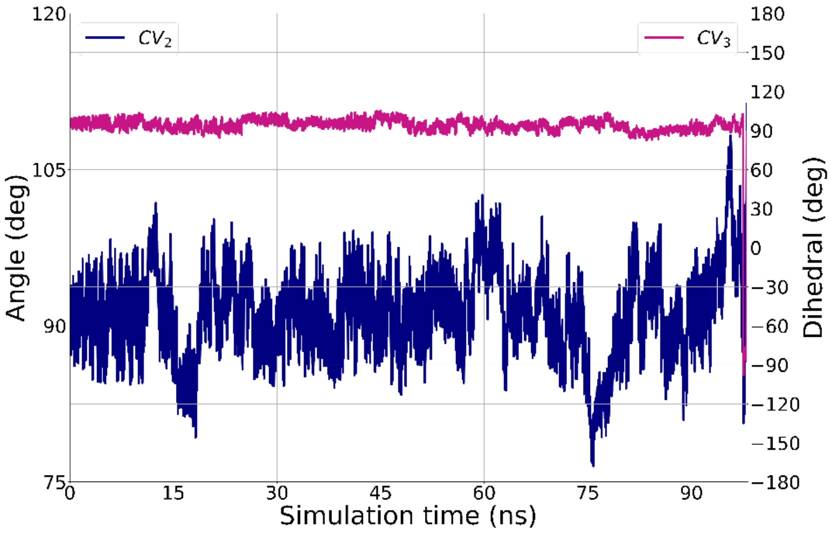

Figure S4. Evolution of CV_2 and CV_3 during a tiotropium unbinding simulation.

Definition of the CV_4 and CV_5.

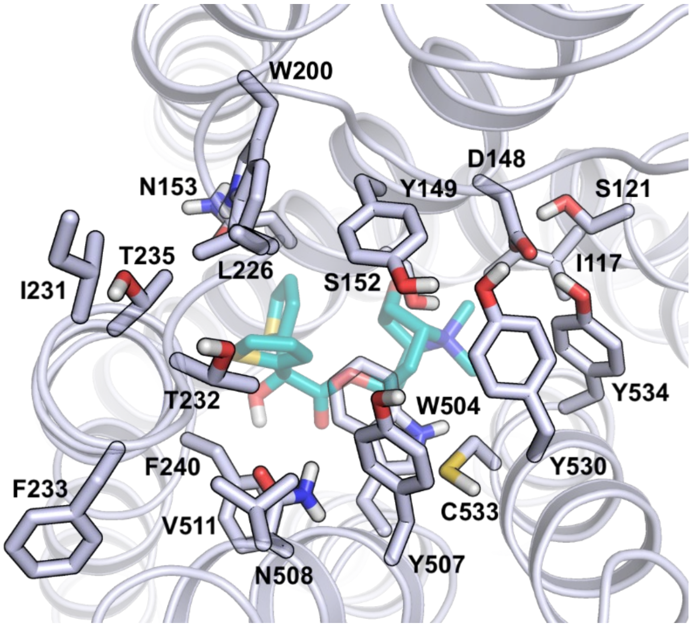

Figure S5. Representation of *h*M3 residues (gray) within 5 Å of tiotropium (transparent dark cyan) used as CV_4. CV_5 is represented by the RMSD of the antagonist under investigation.

Feasibility and optimization of the *conformational flooding* protocol.

Table S2. Log RT_{calc} values (expressed in minutes) for each independent metadynamics of unbinding of tiotropium, **9**, and **1** employing 3 or 4 (a/b) or 5 CVs. Log τ values (expressed in minutes) are also reported as mean ± SEM.

	**3 CVs**			**4 CVs$_a$**			**4 CVs$_b$**			**5 CVs**		
N. rep	tiotropium	**9**	**1**	tiotropium	**9**	**1**	tiotropium	**9**	**1**	tiotropium	**9**	**1**
1	22.8	13.0	8.86	17.9	17.8	9.32	16.7	10.9	8.63	16.8	9.97	4.16
2	21.3	19.1	8.11	23.4	12.9	13.2	16.5	10.8	8.12	11.9	9.67	9.47
3	26.2	22.2	11.0	20.7	17.2	6.74	17.5	11.2	5.16	16.2	10.6	7.78
4	17.3	17.9	6.7	17.8	13.3	5.89	19.5	13.6	6.25	15.7	12.1	6.75
5	25.8	16.5	18.4	14.4	12.5	13.2	12.5	11.3	9.98	13.2	5.79	9.45
6	24.7	15.7	14.5	14.3	13.6	14.0	18.2	14.8	8.13	12.7	9.75	5.99
7	24.4	23.6	6.00	18.7	13.5	10.1	20.0	15.0	8.32	18.1	10.8	8.44
8	18.4	21.9	5.91	21.9	11.9	9.60	17.3	12.0	10.3	13.9	11.0	4.41
9	20.8	22.3	14.3	23.1	12.5	9.04	16.5	15.1	8.04	13.7	9.01	3.87
10	26.5	13.7	13.5	20.1	19.6	10.00	13.9	15.6	7.27	13.8	9.33	7.61
**mean (log τ)**	22.8	18.6	10.7	19.2	14.5	10.1	16.9	13.0	8.02	14.6	9.80	6.79
**SEM**	1.03	1.21	1.36	1.02	0.85	0.85	0.72	0.62	0.49	0.63	0.53	0.67

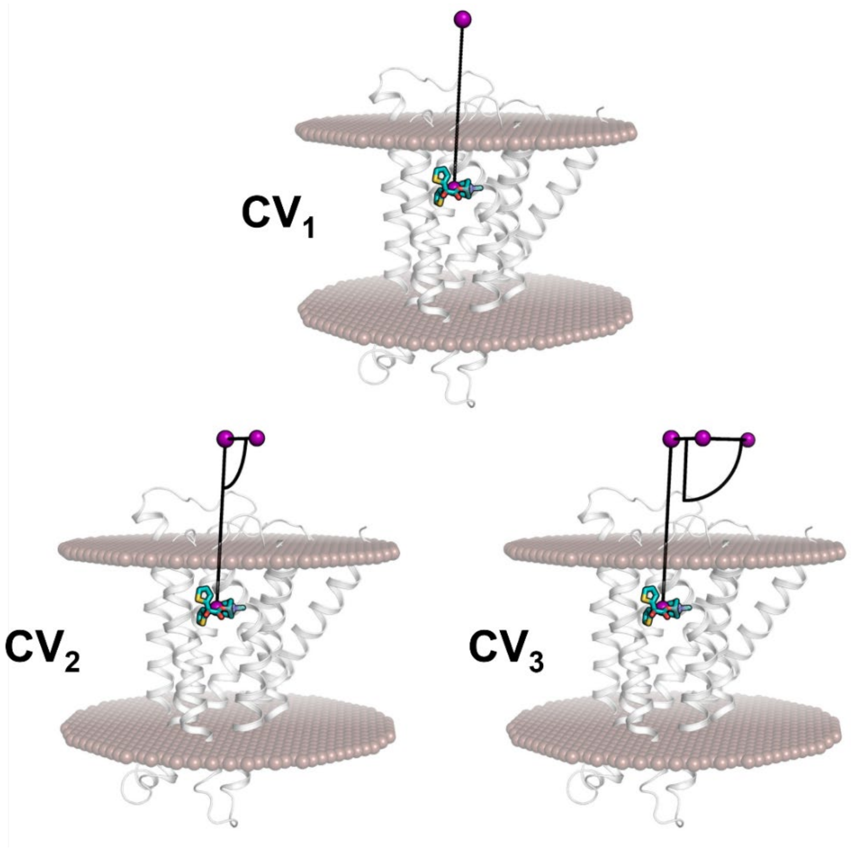

Figure S6. Graphical representations of the 3 alternative CVs employed for *wt*-META-D simulations of tiotropium unbinding from the M3 receptor (dark cyan and gray, respectively) with 5 CVs. CV_1 is the distance between the COM of tiotropium heavy atoms and a point taken in the extracellular side of M3-tiotropium complex. CV_2 represents the angle defined by the two points defined for CV_1 and another vertex also defined on the extracellular side of the M3-tiotropium complex. CV_3 is the dihedral angle defined by the three points used for CV_2 and a point taken on the extracellular side of the M3-tiotropium complex. Definition of these alternative CV_1-CV_3 aims at mimicking the previous intracellular 3 CVs with an opposite direction. The COM of tiotropium heavy atoms and points taken in the extracellular portion of M3-tiotropium complex are depicted with purple spheres. CV_4 is the RMSD of sidechain residues within 5 Å of tiotropium and CV_5 is the RMSD of ligand heavy atoms.

Table S3. Log RT_{calc} values (expressed in minutes) for each independent metadynamics of unbinding of tiotropium, **9**, and **1** employing an alternative set of CVs. Log τ values (expressed in minutes) are also reported as mean ± SEM.

	**alternative 5 CVs**		
N. rep	tiotropium	**9**	**1**
1	13.8	13.9	3.82
2	10.5	17.2	7.43
3	13.5	10.3	3.24
4	14.7	14.1	8.91
5	19.7	12.5	4.98
6	14.6	11.5	3.52
7	14.0	10.1	9.11
8	20.0	12.5	6.33
9	12.2	8.47	5.87
10	16.7	14.1	12.1
**mean (log τ)**	15.0	12.5	6.53
**SEM**	0.96	0.80	0.91

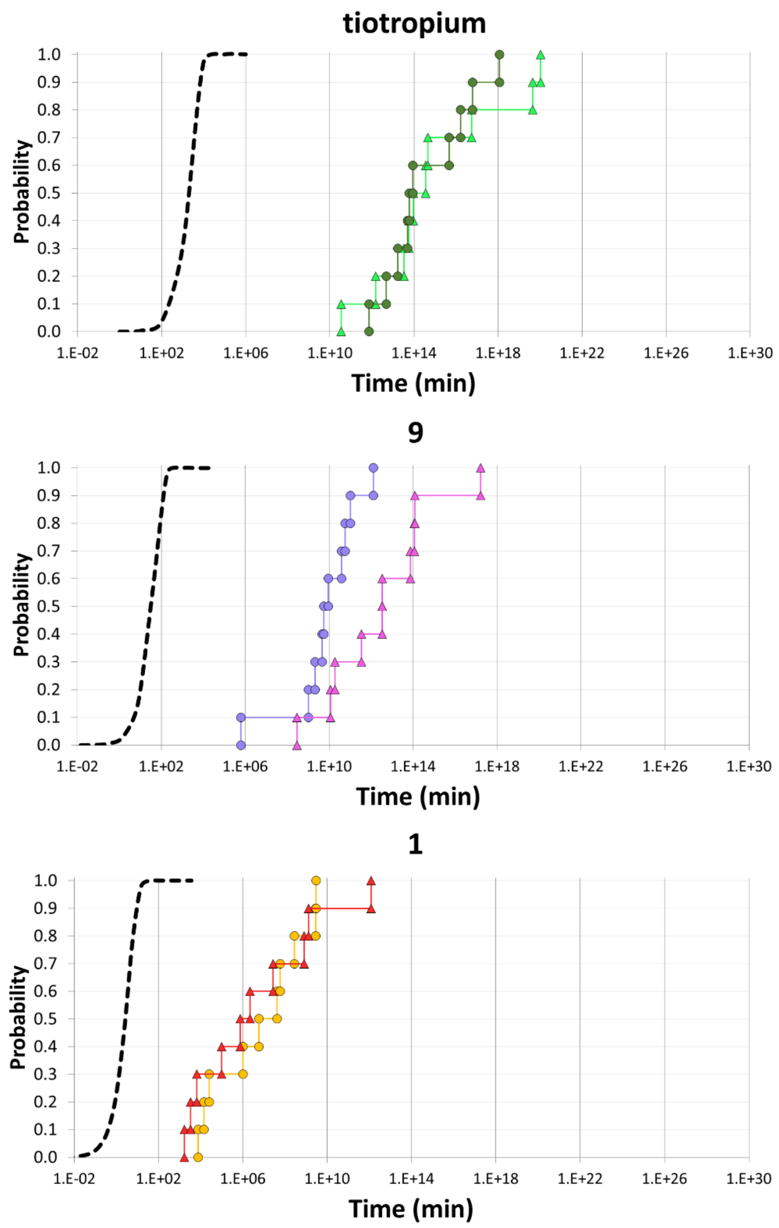

Figure S7. Empirical cumulative distributions (ECDs) derived from unbinding simulations for tiotropium, **9** and **1** with the original 5 CVs (dark green, violet and yellow, respectively) and with an alternative set of 5 CVs in which the first 3 CVs have been modified according to Figure S6 (light green, pink and red ECDs for tiotropium, **9** and **1**, respectively). Dotted black lines represent theoretical Poisson distributions with characteristic parameters corresponding to experimental RTs for tiotropium, **9** and **1**, respectively.

Extension of the *conformational flooding* protocol with 5 CVs to the entire dataset.

Table S4. Log RT_{calc} values (expressed in minutes) for each independent metadynamics of unbinding of all the Tautermann dataset[3] employing 5 CVs. Log τ values (expressed in minutes) are also reported as mean ± SEM.

	5 CVs									
N. rep	tiotropium	**6**	**8**	NMS	**5**	**3**	**9**	**7**	**10**	**1**
1	16.8	10.0	16.5	11.1	11.0	13.1	9.97	13.1	10.9	4.16
2	11.9	16.4	9.72	5.80	12.6	6.10	9.67	11.5	12.3	9.47
3	16.2	13.9	13.8	7.90	15.3	7.70	10.6	9.66	13.8	7.78
4	15.7	12.5	13.6	10.9	12.5	17.1	12.1	12.3	13.9	6.75
5	13.2	14.7	14.0	6.83	8.90	13.1	5.79	6.53	16.9	9.45
6	12.7	12.1	15.8	5.62	13.4	4.98	9.75	12.2	8.02	5.99
7	18.1	15.4	12.0	7.63	14.5	9.24	10.8	6.86	10.8	8.44
8	13.9	11.5	12.0	6.24	15.0	13.4	11.0	8.21	9.41	4.41
9	13.7	15.5	12.2	7.79	13.1	11.4	9.01	8.41	5.84	3.87
10	13.8	12.9	16.2	7.14	6.73	10.9	9.33	8.61	6.67	7.61
**mean (log τ)**	14.6	13.5	13.6	7.70	12.3	10.7	9.80	9.74	10.9	6.79
**SEM**	0.63	0.64	0.69	0.61	0.87	1.18	0.53	0.75	1.10	0.67

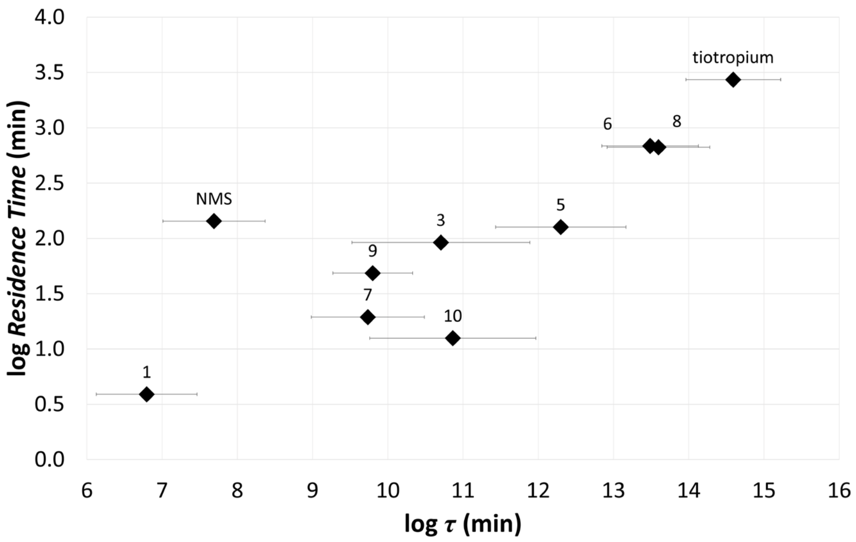

Figure S8. Log experimental RT vs log τ values (expressed in minutes) for the Tautermann dataset of M3 antagonist. Bars represent the SEM over 10 metadynamics replicas.

Binding mode of ipratropium into M3 receptor.

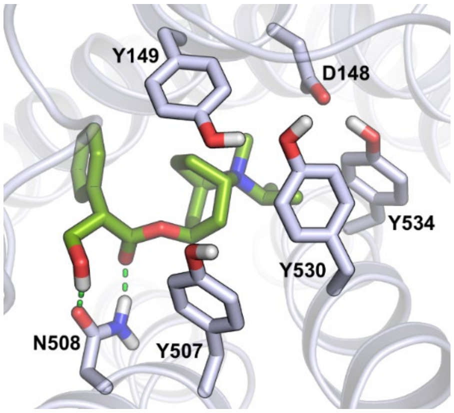

Figure S9. Molecular model of ipratropium (green) within *h*M3 orthosteric binding site (gray). Hydrogen bonds involving the ligand and Asn508$^{6.52}$ are depicted with green dashed lines.

Extension of the *conformational flooding* protocol with 5 CVs to ipratropium.

Table S5. Log RT_{calc} (expressed in minutes) values for each independent metadynamics of unbinding of ipratropium employing 5 CVs.[4] Log τ values (expressed in minutes) are also reported as mean ± SEM.

	**5 CVs**
N. rep	ipratropium
1	11.2
2	13.3
3	13.5
4	9.53
5	8.54
6	7.45
7	4.06
8	2.32
9	9.31
10	6.11

**mean (log τ)**	8.53
**SEM**	1.16

Binding mode of BS46 and darifenacin into M3.

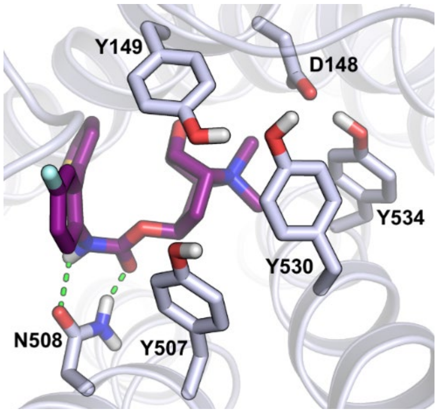

Figure S10. Molecular model of BS46 (purple) within *h*M3 orthosteric binding site (gray). Hydrogen bonds involving the ligand and Asn508$^{6.52}$ are depicted with green dashed lines.

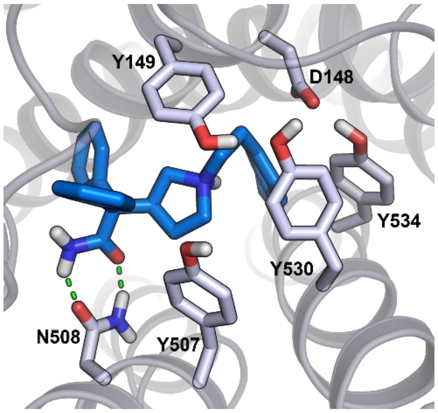

Figure S11. Molecular model of darifenacin (blue) within *h*M3 orthosteric binding site (gray). Hydrogen bonds involving the ligand and Asn508$^{6.52}$ are depicted with green dashed lines.

Extension of the *conformational flooding* protocol with 5 CVs to BS46 and darifenacin.

Table S6. Log RT_{calc} (expressed in minutes) values for each independent metadynamics of unbinding of darifenacin and BS46 with 5 CVs.[5] Log τ values (expressed in minutes) are also reported as mean ± SEM.

	**5 CVs**	
N. rep	BS46	darifenacin
1	12.6	9.82
2	13.4	7.98
3	11.9	8.82
4	8.85	10.6
5	11.3	13.0
6	21.2	11.6
7	14.1	12.4
8	14.9	11.7
9	13.1	7.73
10	8.94	10.7
**mean (log τ)**	13.0	10.4
**SEM**	1.11	0.58

Application of the t_{META-D} protocol with 3 CVs to the entire dataset.

Table S7. t_{META-D} values (expressed in nanoseconds) for each independent metadynamics of unbinbing of Tautermann dataset[3] employing 3 CVs. Averaged t_{META-D} values are also reported as mean ± SEM.

	3 CVs									
N. rep	tiotropium	**6**	**8**	NMS	**5**	**3**	**9**	**7**	**10**	**1**
1	26.4	24.8	21.1	14.5	20.2	34.3	19.6	20.3	12.1	16.5
2	27.3	23.0	20.2	17.8	15.4	20.9	25.1	11.1	19.0	15.5
3	26.2	26.0	27.0	22.9	17.6	24.4	21.5	12.7	24.1	10.0
4	19.5	18.7	19.7	18.1	22.9	19.7	20.9	15.6	15.0	10.9
5	24.2	34.3	21.0	15.3	22.8	27.3	23.1	12.9	14.3	12.4
6	27.8	22.7	24.2	12.2	24.3	26.6	18.3	18.0	16.0	12.3
7	16.4	30.1	33.4	14.8	19.8	17.3	24.1	11.6	17.5	11.1
8	21.0	31.9	19.0	25.9	11.8	18.0	36.7	14.7	14.8	14.1
9	28.1	23.4	30.1	16.3	20.8	18.0	21.6	15.8	18.7	10.2
10	42.1	27.2	22.7	11.8	24.0	33.2	11.2	17.4	17.1	12.8
**mean**	25.9	26.2	23.8	17.0	20.0	24.0	22.2	15.0	16.9	12.6
**SEM**	2.19	1.50	1.53	1.42	1.27	1.98	2.02	0.95	1.05	0.70

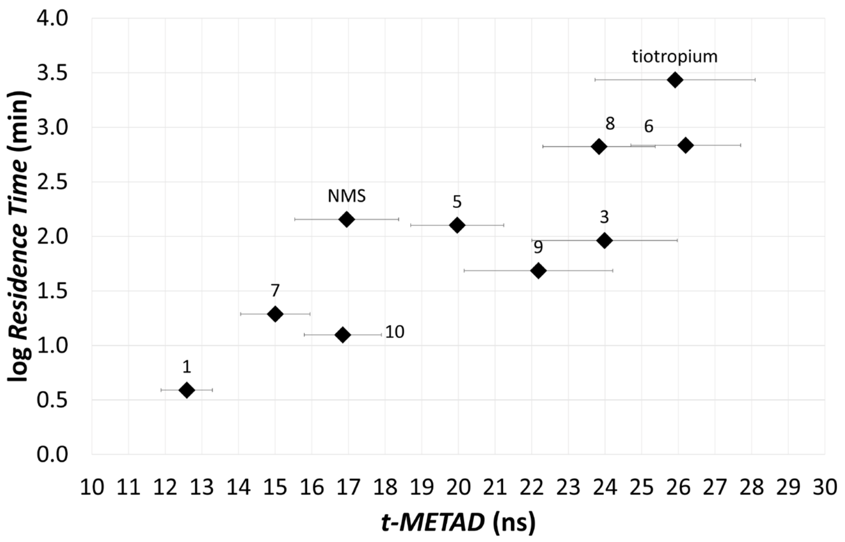

Figure S12. Log experimental RT vs $t_{META\text{-}D}$values (expressed in minutes and nanoseconds, respectively) for the Tautermann dataset of M3 antagonist. Bars represent the SEM over 10 metadynamics replicas.

Extension of the $t_{META\text{-}D}$ protocol with 3 CVs to ipratropium.

Table S8. $t_{META\text{-}D}$ values (expressed in nanoseconds) for each independent metadynamics of unbinding of ipratropium[4] employing 3 CVs. Averaged $t_{META\text{-}D}$ values are also reported as mean ± SEM.

	**3 CVs**
N. rep	ipratropium
1	10.0
2	13.4
3	10.5
4	10.5
5	17.0
6	11.6
7	12.1
8	10.6
9	10.0
10	13.9

**mean**	12.0
**SEM**	0.71

Extension of the t_{META-D} protocol with 3 CVs to BS46 and darifenacin.

Table S9. t_{META-D} values (expressed in nanoseconds) for each independent metadynamics of unbinding of BS46 and darifenacin employing 3 CVs.[5] Averaged t_{META-D} values are also reported as mean ± SEM.

	3 CVs	
N. rep	BS46	darifenacin
1	19.5	25.5
2	22.6	16.3
3	23.7	10.3
4	21.4	18.3
5	20.6	17.7
6	18.6	16.3
7	27.7	25.9
8	29.3	18.0
9	25.9	25.4
10	21.5	13.5

**mean**	23.1	18.7
**SEM**	1.12	1.68

Details on the homology modelling procedure for *h*M3.

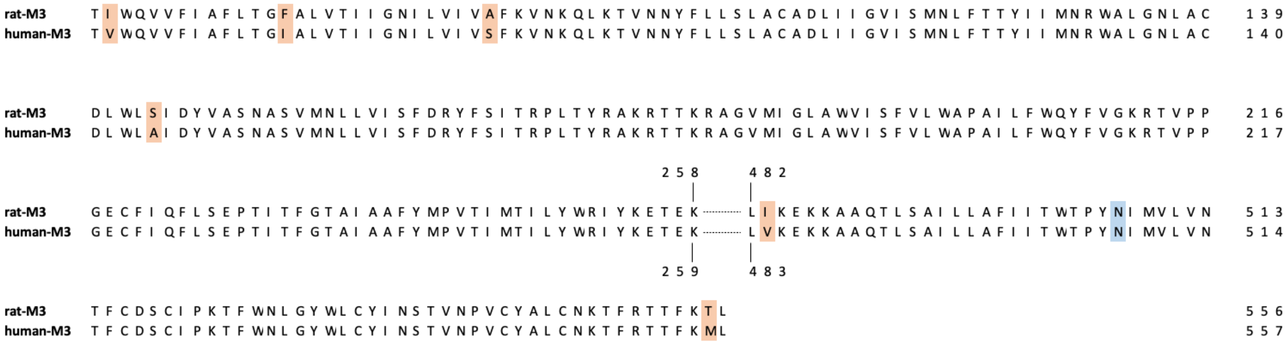

Figure S13. Sequence alignment of *rat* and *human* M3 receptors: mutated residues are highlighted in orange and the key residue Asn508$^{6.52}$ is colored in blue. The intracellular portion was removed from both the sequences due to the absence of a reliable template for modeling.

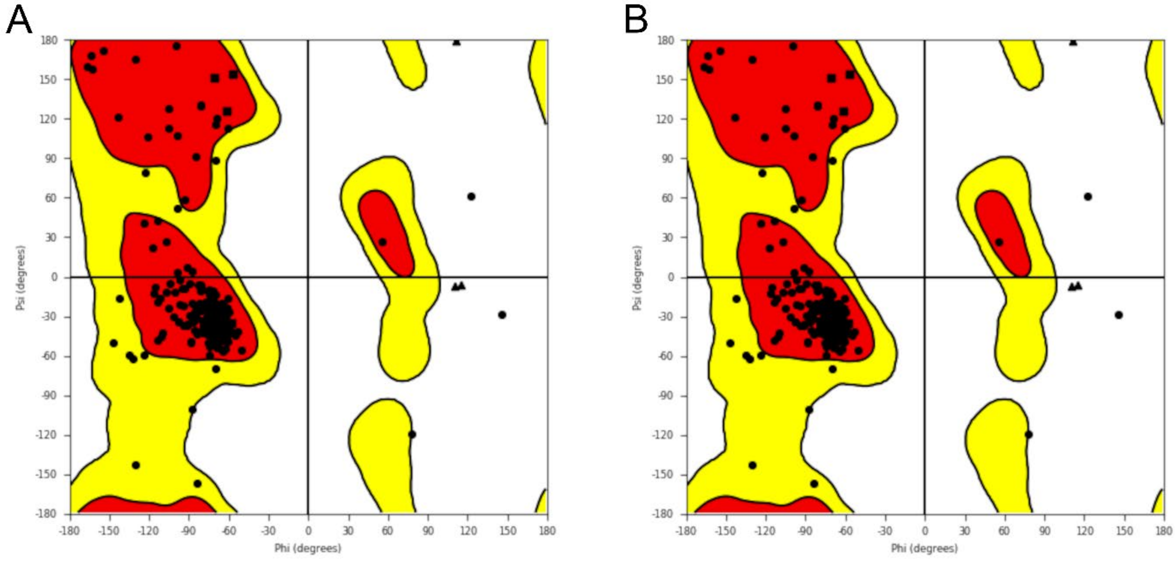

Figure S14. Ramachandran plot of *rat* M3 model (A) in comparison with the *human* one (B).

References

[1] Glide, Schrödinger, LLC, New York, NY, 2021.

[2] Friesner, R. A.; Banks, J. L.; Murphy, R. B.; Halgren, T. A.; Klicic, J. J.; Mainz, D. T.; Repasky, M. P.; Knoll, E. H.; Shelley, M.; Perry, J. K.; Shaw, D. E.; Francis, P.; Shenkin, P. S. Glide: a new approach for rapid, accurate docking and scoring. 1. Method and assessment of docking accuracy. *J. Med. Chem*. **2004**, *47*, 1739−1749.

[3] Tautermann, C. S.; Kiechle, T.; Seeliger, D.; Diehl, S.; Wex, E.; Banholzer, R.; Gantner, F.; Pieper, M. P.; Casarosa, P. Molecular basis for the long duration of action and kinetic selectivity of tiotropium for the muscarinic M3 receptor. *J. Med. Chem.* **2013**, *56*, 8746–8756.

[4] Glossop, P. A.; Watson, C. A.; Price, D. A.; Bunnage, M. E.; Middleton, D. S.; Wood, A.; James, K.; Roberts, D.; Strang, R. S.; Yeadon, M.; Perros-Huguet, C.; Clarke, N. P.; Trevethick, M. A.; Machin, I.; Stuart, E. F.; Evans, S. M.; Harrison, A. C.; Fairman, D. A.; Agoram, B.; Burrows, J. L.; Feeder, N.; Fulton, C. K.; Dillon, B. R.; Entwistle, D. A.; Spence, F. J. Inhalation by design: novel tertiary amine muscarinic M3 receptor antagonists with slow off-rate binding kinetics for inhaled once-daily treatment of chronic obstructive pulmonary disease. *J. Med. Chem.* **2011**, *54*, 6888−6904.

[5] Liu, H.; Hofmann, J.; Fish, I.; Schaake, B.; Eitel, K.; Bartuschat, A.; Kaindl, J.; Rampp, H.; Banerjee, A.; Hübner, H.; Clark, M. J.; Vincent, S. G.; Fisher, J. T.; Heinrich, M. R.; Hirata, K.; Liu, X.; Sunahara, R. K.; Shoichet, B. K.; Kobilka, B. K.; Gmeiner, P. Structure-guided development of selective M3 muscarinic acetylcholine receptor antagonists. *Proc. Natl. Acad. Sci. U. S. A.* **2018**, *115*, 12046–12050.
